# An alternative approach to nucleic acid memory

**DOI:** 10.1038/s41467-021-22277-y

**Published:** 2021-04-22

**Authors:** George D. Dickinson, Golam Md Mortuza, William Clay, Luca Piantanida, Christopher M. Green, Chad Watson, Eric J. Hayden, Tim Andersen, Wan Kuang, Elton Graugnard, Reza Zadegan, William L. Hughes

**Affiliations:** 1 Micron School of Materials Science and Engineering, Boise State University, Boise, ID USA; 2 Department of Computer Science, Boise State University, Boise, ID USA; 3 Department of Biological Sciences, Boise State University, Boise, ID USA; 4 Department of Electrical and Computer Engineering, Boise State University, Boise, ID USA; 5 Present Address: Center for Bio/Molecular Science and Engineering, U.S. Naval Research Laboratory, Washington, DC USA; 6 Present Address: Department of Nanoengineering, Joint School of Nanoscience and Nanoengineering, North Carolina A&T State University, Greensboro, NC USA

**Keywords:** DNA computing and cryptography, Information storage, DNA nanostructures, Super-resolution microscopy

## Abstract

DNA is a compelling alternative to non-volatile information storage technologies due to its information density, stability, and energy efficiency. Previous studies have used artificially synthesized DNA to store data and automated next-generation sequencing to read it back. Here, we report digital Nucleic Acid Memory (dNAM) for applications that require a limited amount of data to have high information density, redundancy, and copy number. In dNAM, data is encoded by selecting combinations of single-stranded DNA with (1) or without (0) docking-site domains. When self-assembled with scaffold DNA, staple strands form DNA origami breadboards. Information encoded into the breadboards is read by monitoring the binding of fluorescent imager probes using DNA-PAINT super-resolution microscopy. To enhance data retention, a multi-layer error correction scheme that combines fountain and bi-level parity codes is used. As a prototype, fifteen origami encoded with ‘Data is in our DNA!\n’ are analyzed. Each origami encodes unique data-droplet, index, orientation, and error-correction information. The error-correction algorithms fully recover the message when individual docking sites, or entire origami, are missing. Unlike other approaches to DNA-based data storage, reading dNAM does not require sequencing. As such, it offers an additional path to explore the advantages and disadvantages of DNA as an emerging memory material.

## Abstract (web summary)

Encoding data in DNA is a promising approach to high density data storage. Here the authors present a prototype sequencing-free method that uses the spatial orientation of DNA strands with super-resolution microscopy readout.

## Introduction

As outlined by the Semiconductor Research Corporation, memory materials are approaching their physical and economic limits^1,2^. Motivated by the rapid growth of the global datasphere^3^, and its environmental impacts, new non-volatile memory materials are needed. As a sustainable alternative, DNA is a viable option because of its information density, significant retention time, and low energy of operation^4^. While synthesis and sequencing cost curves drive innovations in the field, divergent approaches to nucleic acid memory (NAM) have been constrained because of the ease of using sequencing to recover stored digital information^5–13^^.^

**Fig. 1:**
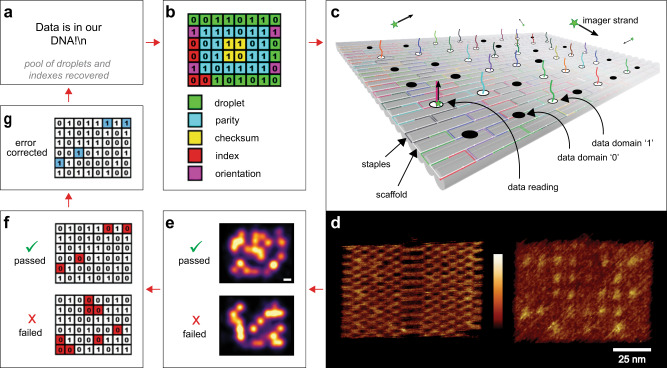
Binary dNAM overview. The test message (**a**) for optically reading dNAM was ‘Data is in our DNA!’. The message was encoded and then synthesized into 15 dNAM origami. For clarity, only one of the 15 designs is shown in (**b**). The data domain colors correspond to their bit values as follows: droplet (green), parity (blue), checksum (yellow), index (red), and orientation (magenta). Site-specific localization is enabled by extending or not-extending the structural staple strands of the origami to create physical representations of 1s and 0s. The presence, absence, and identity of a data strand’s docking sequence defines the state of each data strand and is assessed by monitoring the binding of data imager strands via DNA-PAINT in (**c**). AFM images of an origami nanostructure are depicted in (**d**), with both the expected raft honeycomb structure (left) and data strands (right) visible. The scale bar is 25 nmin the AFM images and the color scale ranges from 0–1 nm in height. To ‘read’ the encoded message, 4 μL of the DNA origami mixture, containing 0.33 nM of each origami, was imaged via DNA-PAINT. Two representative origami cropped from the final rendered image are shown in (**e**), scale bar, 10 nm. All structures identified as origami in the rendered image were converted to a matrix of 1’s and 0’s corresponding to the pattern of localizations seen at each data domain in (**f**). The red boxes in (**f**) now indicate errors. The decoding algorithm performed error correction where possible in (**g**) and successfully retrieved the entire message when sufficient data droplets and indexes were recovered in (**a**). The blue boxes in (**g**) now indicate corrected errors.

Here, we report digital Nucleic Acid Memory (dNAM) as an alternative to sequencer-based DNA memory. Inspired by progress in DNA nanotechnology^14^, dNAM uses advancements in super-resolution microscopy (SRM)^15^ to access digital data stored in short oligonucleotide strands that are held together for imaging using DNA origami. In dNAM, non-volatile information is digitally encoded into specific combinations of single-stranded DNA, commonly known as staple strands, that can form DNA origami nanostructures when combined with a scaffold strand. When formed into origami, the staple strands are arranged at addressable locations (Fig. 1) that define an indexed matrix of digital information. This site-specific localization of digital information is enabled by designing staple strands with nucleotides that extend from the origami. Extended staple strands have two domains: the first domain forms a sequence-specific double helix with the scaffold and determines the address of the data within the origami; the second domain extends above the origami and, if present, provides a docking site for fluorescently labeled single-stranded DNA imager strands. Binary states are defined by the presence (1) or absence (0) of the data domain, which is read with a super-resolution microscopy technique called DNA-Points Accumulation for Imaging in Nanoscale Topography (DNA-PAINT)^16^. Unique patterns of binary data are encoded by selecting which staple strands have, or do not have, data domains. As an integrated memory platform, data is entered into dNAM when the staple strands encoding 1 or 0 are selected for each addressable site. The staple strands are then stored directly, or self-assembled into DNA origami and stored. Editing data is achieved by replacing specific strands or the entire content of a stored structure. To read the data, the origami is optically imaged below the diffraction limit of light using DNA-PAINT (Fig. S1).

Key design features of dNAM, that ensure error-free data recovery, are our error-correcting algorithms. Detection of individual DNA molecules using DNA-PAINT is routinely limited by incomplete staple strand incorporation, defective imager strands, fluorophore bleaching, and/or background fluorescence^17^. Although it is possible to improve the signal-to-noise ratio by averaging multiple images of identical structures^17^, this approach comes at a significant cost to the read speed and information density. To overcome these challenges, we created dNAM-specific information encoding and decoding algorithms that combine fountain codes with a custom, bi-level, parity-based, and orientation-invariant error detection scheme. Fountain codes enable transmission of data over noisy channels^18^. They work by dividing a data file into smaller units called droplets and then sending the droplets at random to a receiver. Droplets can be read in any order and still be decoded to recover the original file^19^, so long as a sufficient number of droplets are sent to ensure that the entire file is received. We encode each droplet onto a single origami and add additional bits of information for error correction to ensure that individual droplets will be recovered, in the presence of high noise, from individual DNA origami. Together, the error-correction and fountain codes increase the probability that the message is fully recovered while reducing the number of origami that must be observed.

In this report, we describe a working prototype of dNAM. As a proof of concept, we encoded the message ‘Data is in our DNA!\n’ into origami and recovered the message using DNA-PAINT. We divided the message into 15 digital droplets, each encoded by a separately synthesized origami with addressable staple strands that space out data domains approximately 10 nm apart. A single DNA-PAINT recording recovered the message from 20 fmoles of origami, with approximately 750 origami needing to be read to reach a 100% probability of full data retrieval. By combining the spatial control of DNA nanotechnology with our error-correction algorithms, we demonstrate dNAM as an alternative approach to prototyping DNA-based storage for applications that require a limited amount of data to have high information density, redundancy, and copy number.

## Results

### Recovery of a message encoded into dNAM

To test our dNAM concept, we encoded the message ‘Data is in our DNA!\n’ into 15 distinct DNA origami nanostructures (Fig. 1a). Each origami was designed with a unique 6 × 8 data matrix that was generated by our encoding algorithm with data domains positioned ~10 nm apart. For encoding purposes, the message was converted to binary code (ASCII) and then segmented into 15 overlapping data droplets that were each 16 bits. Inspired in part by digital encoding formats like QR-codes, the 48 addressable sites on each origami were used to encode one of the 16-bit data droplets, as well as information used to ensure the recovery of each data droplet. Specifically, each origami was designed to contain a 4-bit binary index (0000–1110), twenty bits for parity checks, four bits for checksums, and four bits allocated as orientation markers (Fig. 1b). To fully recover the encoded message, we then synthesized each origami separately and deposited an approximately equal mixture of all 15 designs (~20 fmoles of total origami) onto a glass coverslip. The data domains were accessible for binding via fluorescently labeled imager probes because they faced the bulk solution and not the coverslip (Fig. 1c). High-resolution atomic force microscopy (AFM) was used in tapping mode to confirm the structural integrity of the origami and the presence of the data domains (Fig. 1d). 40,000 frames from a single field of view were recorded using DNA-PAINT (~4500 origami identified in 2982 µm^2^). The super-resolution images of the hybridized imager strands were then reconstructed from blinking events identified in the recording to map the positions of the data domains on each origami (Fig. 1e). Using a custom localization processing algorithm, the signals were translated to a 6 × 8 grid and converted back to a 48-bit binary string—which was passed to the decoding algorithm for error correction, droplet recovery, and message reconstruction (Fig. 1f, g). The process enabled successful recovery of the dNAM encoded message from a single super-resolution recording.

### Quality control of dNAM

**Fig. 2:**
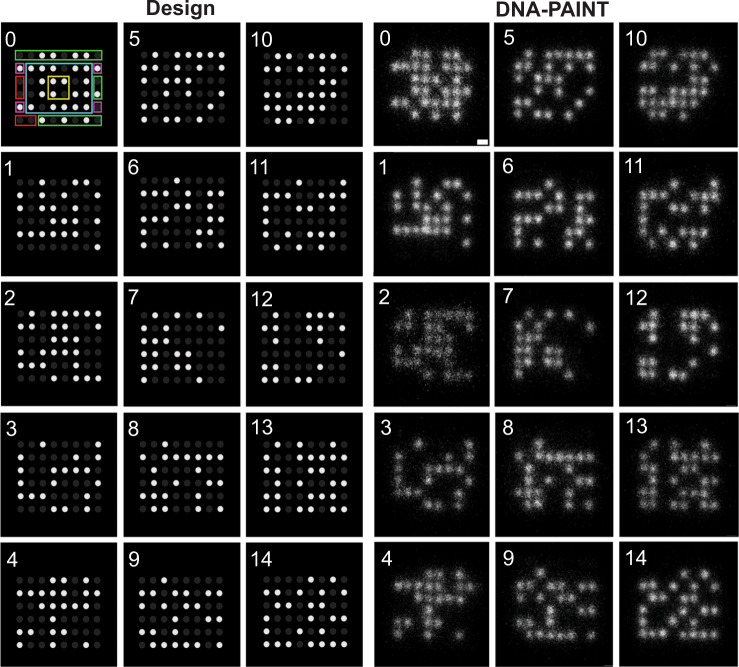
DNA-PAINT imaging of dNAM indicates all sites are recovered in a single read. dNAM origami from a DNA-PAINT recording were identified and classified by aligning and template matching them with the 15 design matrixes (Design) in which all potential docking sites are shown. Filled circles indicate sites encoded ‘0’ (dark gray) or ‘1’ (white). Colored boxes indicate the regions of the matrixes used for the droplet (green), parity (blue), checksum (yellow), index (red), and orientation (magenta). For clarity, only the first design image includes the colored matrix sites. Averaged images of 4560 randomly selected origami, grouped by index, are depicted (DNA-PAINT). Scale bar, 10 nm.

We evaluated all of the origami structures using AFM to confirm that the 15 different designs were successfully synthesized, with their data domains in the correct location. Automated image processing algorithms were developed to identify, orient, and average multiple images of each origami from the DNA-PAINT recording of the mixture (Fig. 2). Although the edges of the origami were more sensitive to data strand insertion failures (Fig. S2), the results confirmed that all of the data domains, in each of the origami designs, were detectable in each of the three separate experiments. The AFM images further confirmed that the general shapes of all 15 origami designs were as expected with properly positioned data domains (Fig. 1d, Fig. S3). The results indicate that the extended staple strands do not prevent the synthesis of the 15 unique origami designs.

### Further AFM analysis of dNAM origami

**Fig. 3:**
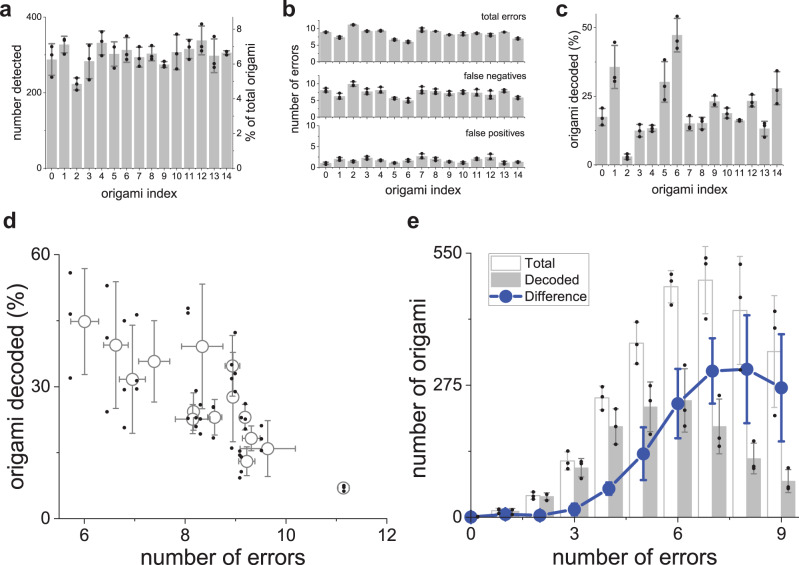
All 15 dNAM data strings were recovered from a single read. **(a)** plots the numbers of each origami index observed in a single recording, based on template matching. The mean counts are shown as gray bars, with the percentage of the total origami indicated on the secondary axis. In (**b**), the mean number of total errors (top) for each structure is shown, based on template matching. The same errors are also shown after being grouped into false negatives (middle) and false positives (bottom). (**c**) depicts the percent of origami passed to the decoding algorithm that had both their indexes and data strings correctly identified. In (**d**), the percentage of each origami decoded is plotted against the mean number of errors for each structure. (**e**) shows histograms of the total mean numbers of errors found in origami identified by template matching (open bars) and the decoding algorithm (gray bars). The difference between the two is plotted in blue. Mean values for three experiments are depicted in all graphs, error bars indicate ±SD. Individual data points are plotted as small black circles.

As an additional quality control step, we used AFM to examine origami deposited onto a glass coverslip immediately following SRM imaging. We were not able to resolve individual docking sites in these images, most likely due to the increased roughness of glass, as compared to mica. However, it was possible to count the number of origami in a field of view for comparison with SRM. The densities of origami estimated from the images were 2.4 and 1.4 origami/µm^2^ for AFM and SRM, respectively, suggesting that ~60% of the total origami deposited on glass have their data domains facing away from the coverslip and available for imager strand binding. To further investigate the variance in error rates between origami designs, we resynthesized the most error-prone origami (origami index 2). DNA-PAINT imaging indicated that the new batch showed 9.7 ± 2 false-negative errors per origami, consistent with the original experiment, while the second batch showed 7.1 ± 2 false-negative errors (Fig. 3). This suggests that at least a portion of the variance in error rates is independent of origami design and may be caused by variations in mixing, folding, and purification conditions.

### Data encoding/decoding strategy for dNAM

Our encoding approach added 24 error-correction bits of data to every origami structure so that data droplets can be determined from individual origami even when data domains are incorrectly resolved, and the entire message recovered if some droplets are missed entirely. To evaluate the performance of the decoding algorithm, we examined the frequency and types of errors in the DNA-PAINT images and the effect of these errors on our decoding outcomes. We used a template matching strategy where each of the 15 origami designs was considered a template, and each individual origami in the field of view was compared to these designs to find the best match. We identified the total number of origami that matched or did not match, each design (Fig. 3a, b). We then determined the number of each design identified by the decoding algorithm when recovering the message (Fig. 3c): a process independent of template matching and blind to the droplet data contained in the DNA origami. We observed a clear negative correlation between the number of errors detected in a specific design and the number of corresponding origami that were successfully decoded by the algorithm (Fig. 3d). The results indicate that, even though there was a low relative abundance of several origami in the deposition mixture (particularly origami index 2) and a mean of 7.3 ± 1.2 false errors per origami across the different designs, our error-correction scheme enabled successful message recovery. False positives were much less common in our experiments, with a mean of 1.7 ± 0.5 (Fig. 3b). Furthermore, the mean number of errors overcome by the decoding algorithm (5.5 ± 0.1) was lower than the mean number of errors observed across all the origami (7.7 ± 0.1), demonstrating the challenge of decoding origami when several fluorescent signals are missing (Fig. 3e). Nevertheless, the ability of our data encoding and decoding strategy to recover the message despite errors in individual origami is promising, and the results provide useful guidelines for evaluating and optimizing origami performance for future dNAM designs.

### Sampling analysis of dNAM

**Fig. 4:**
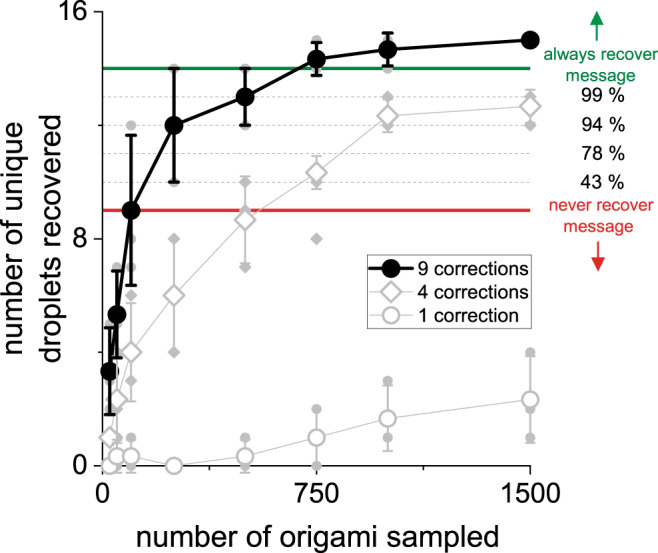
Number of dNAM origami required to recover the message. The mean number of unique dNAM origami correctly decoded for randomly selected subsamples of decoded binary strings are shown. The analysis was broken out by the number of errors corrected for each origami, three examples are plotted (1, 4, and 9). Black filled circles depict the mean results for nine error corrections, which is the ‘maximum allowable number of errors’ parameter used in the decoding algorithm for all other analysis reported here. The horizontal lines indicate the probability of recovering the message with different numbers of unique droplets. With fourteen or more droplets, the message should always be recovered (thick green line, and above indicates 100% chance of recovery) and with nine or fewer droplets the message will never be recovered (thick red line and below indicates 0% chance of recovery). Mean values for three experiments are shown. Error bars indicate ±SD. Individual data points are plotted behind as smaller gray symbols.

Given the observed frequency of missing data points, we then used a random sampling approach to determine the number of origami needed to decode the ‘Data is in our DNA!\n’ message under our experimental conditions. We started with all the decoded binary output strings that were obtained from the single-field-of-view recordings and took random subsamples of 50–3000 binary strings. We passed each random subsample of strings through the decoding algorithm and determined the number of droplets that were recovered (Fig. 4). Based on the algorithmic settings used in the experiment, we found that only ~750 successfully decoded origami were needed to recover the message with near 100% probability. This number is largely driven by the presence of origami in our sample that were prone to high error rates and thus rarely decoded correctly (i.e., origami index 2).

### Simulations of dNAM

**Fig. 5:**
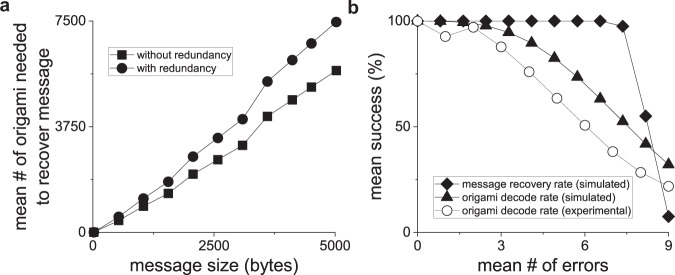
dNAM origami and message recovery rates in the presence of increasing errors. Simulations were performed to determine the theoretical success rates for correctly decoding individual dNAM origami and recovering encoded messages. In (**a**), the mean number of dNAM origami needed to successfully recover messages of increasing length with (circles) or without (squares) redundant bits are plotted. In (**b**), the mean success for recovering both individual origami (triangles) and the entire message (diamonds) are plotted against the mean number of errors per origami (errors were randomly generated for simulated data). Simulation recovery rates are averages of all message sizes tested (160 to 12,800 bits). For comparison, the mean success rate for experimental data is also plotted (open circles). For experimental data, the mean success was estimated by comparing the decode algorithm’s results with that of the template-matching algorithm. All simulations were repeated 40 times. Experimental data were derived from 3 independent DNA-PAINT recordings.

Simulations were run to determine the size efficiency of the encoding scheme, as well as its ability to recover from errors. As shown in Fig. 5a, the number of origami required to encode a message of length *n* increases roughly at a linear rate up to *n* = 5000 bytes of data. Larger message sizes require more bits to be devoted to indexing, decreasing the number of available data bits per origami—creating a practical limit of 64 kB of data for the prototype described in this work. This limit can be increased by increasing the number of bits per origami. To determine the ability of the decoding and error correction algorithm to recover information in the presence of increasing error rates, in silico origami that encoded randomly generated data were subjected to increasing bit error rates. The decoding algorithm robustly recovers the entire message for all tested message sizes when the average number of errors per origami is less than 7.4 (Fig. 5b). At 7.4 errors per origami, the message recovery rate drops to 97.5%, and as expected decreases rapidly with higher error rates (55% recovery at 8.2 errors per origami, and 7.5% at 9 errors per origami). An important feature of our algorithm is that the origami recovery rate can be low (as low as 63% in these experiments) and still recover the entire message 100% of the time.

## Discussion

Our results demonstrate a proof of concept for writing and reading digital information encoded in oligonucleotides. Because of the durability of DNA, dNAM has long-term future potential for archival information storage. Currently, the most widely used material for this purpose is magnetic tape. Recent advancements in tape report a two-dimensional areal information density up to 31 Gbit/cm^2^^20^, though the current commercially available material typically has lower density^8^. Although relevant only for reading throughput, not storage, the information density of tape can be compared to the dNAM origami, which contains data domains spaced at 10 nm intervals to achieve an areal density of about 1000 Gbit/cm^2^. After accounting for using ~2/3 of the bits for indexing and error correction, this results in an areal data density of 330 Gbit/cm^2^. It is possible to increase dNAM areal density by placing a data domain at every turn in the DNA helix (~3.5 nm spacing), a distance that has been resolved by SRM^21^. Other avenues to increasing density are also available, such as previously reported multiplexing techniques with multiple fluorophores and orthogonal binding sequences with different binding kinetics^22^, and incorporation of each of these approaches is expected to impact reading throughput. In terms of durability, typical magnetic tape lasts for 10–30 years, while double-stranded DNA is estimated to be stable for millions of years under optimal environmental conditions^7^.

With our optical microscope setup and origami deposition protocol, we can image the 7500 unique origami designs needed to store 5 kB of data (Fig. 5), albeit in several recordings. We conservatively estimate it would take ~30 recordings to ensure a 100% probability of successful data recovery given our current error rates. To efficiently handle larger datasets, it is necessary to improve the data capacity of individual origami, which will allow a larger range of indexing values and increase the proportion of bits dedicated to the data as compared to indexing, error-correction, and orientation. This could be achieved by engineering larger origami or by increasing data density—either by placing data sites closer together or by using multiplexing techniques to augment bit depth at each site (see SI, Supplemental Calculations).

Our results also indicate that advancements in origami-based information storage and reading will require a coordinated effort between improvements in origami synthesis, substrate deposition, DNA-PAINT, and coding algorithms. For example, our subsampling approach (Fig. 4) showed that a decoding algorithm that corrected up to nine errors easily recovered our entire message, while algorithms that corrected only five or fewer errors are much less computationally expensive but rarely recovered our full message. This makes sense, given that most of the origami detected had more than five errors (Fig. 3e). We anticipate that reducing the number of errors by improving origami design and optimizing imager strand performance would allow more efficient algorithms for data recovery, which would, in turn, decrease the number of bits dedicated to error correction and thus increase information density.

Our fountain code algorithm is robust to randomly lost packets of information, as long as the receiver receives *K* + *ε* packets, where *K* is the minimum number of packets required to encode the file under perfect conditions (i.e., *K* is equal to the file size) and *ε* is the number of additional packets received. The probability of being able to decode the file is then (1−*δ*), where *δ* is upper-bounded by 2^−*Kε*^^23^. This equation implies that all things being equal, the larger the file size the greater the likelihood of successfully recovering the file at the receiver. Normally, the transmitter continues to transmit droplets in a fountain code until the receiver acknowledges successful file recovery. In the case of dNAM, this is not possible since the number of droplets must be fixed ahead of time to equal the number of origami. Reducing the error rates, or improving error correction/detection, would have the added benefit of reducing the number of droplets and hence origami discarded by the fountain code. These improvements would make it easier to determine the minimum number of droplets per DNA origami needed to ensure robust file recovery while increasing information density even further.

The lower abundance and higher error rate of origami index 2 (Fig. 3) indicate that some designs have defects that we could not detect by AFM and/or SRM. Careful defect analysis indicates that incorporated but inactive data domains play a greater role in producing errors than unincorporated staple strands^24^. Future dNAM research should focus on sequence optimization to minimize variation in hybridization rates and the formation of off-target structures^25^. It should also include the use of larger DNA origami and increased bit depth through multiplexing.

Future work on dNAM will also need to address scalability if dNAM is to compete with established memory storage systems. In this report, we describe the storage of a small amount of data in order to illustrate the potential of dNAM. Scaling to much larger data sets requires substantial engineering improvements in both write and read speeds (see Fig. S8 and Supplemental Calculations for further comparisons). For writing, the rate-limiting step is the selection of the oligonucleotide data strands. In our lab, we use an EpMotion 5075 liquid-handling system to pipette oligonucleotides. While this machine could handle thousands of sample transfers per day, it limits the write speed to thousands of bits per day as each data strand encodes 1 bit. As far as we are aware, the fastest liquid-transfer system available is the Echo ® 520 Liquid Handler, which is reported by the manufacturer to process ~750,000 samples per day, allowing ~0.1 MB per day for 1-bit data strands. For dNAM to reach write speeds equivalent to tape (hundreds of MB per second) using laboratory hardware, significant increases in either the number of bits per strand and the rate of transfer of samples or the rate at which DNA oligonucleotides can be synthesized will be necessary. While writing information into DNA at a competitive rate is a sincere challenge that is facing the entire DNA-memory field^5^, and is likely to undergo rapid innovation as the market for synthesized DNA increases, the approach we have used here, in which a library of premade oligonucleotides are drawn on, is currently the fastest approach for dNAM.

Due to the inherently parallel nature of DNA-PAINT imaging, the read speed of dNAM is arguably less of a challenge to scale up to deal with large amounts of data. The rate-limiting factors for DNA-PAINT are the camera integration time needed to collect sufficient photons to resolve an emitter and the number of emitters that can be identified in a single frame of a recording. The latest report on DNA-PAINT by Strauss and Jungmann describes a 100-fold speed-up in data collection for origami very similar to those we imaged in dNAM^26^. In their experiments, 5 nm resolution of the binding site was demonstrated with 100 ms camera integration times. Another recent innovation, using deep learning to rapidly identify the centroids of overlapping emitter blink events (Deep-STORM^27^), has been shown to be able to process dense SRM data (~6 emitters/µm^2^). Taken together we estimate that by using densely-deposited dNAM origami^28^ with data strands placed 5 nm apart, an EMCCD camera with a 1024 × 1024 imaging array, the Deep-STORM algorithm, and Straus and Jungmann’s 100-fold speed-up methodology, we could currently collect data at a rate of ~700 MB per day (see SI, Supplemental Calculations). Further improvements in reading speed could be achieved by increasing the imaging array area—via larger sensors or multiple cameras and using multicolored probes or three-dimensional information to collect multiple bits worth of data simultaneously from one site. Our hope is that this dNAM prototype will motivate this work and more.

DNA is an emerging material for data storage due to its high information density, high durability, low energy of operation, and the declining costs of synthesis^1^. The traditional approach in the field is to design and synthesize unique oligonucleotides that encode data directly into their sequence. This data is recovered by reading the pool of oligonucleotides using sequencing. In contrast, dNAM takes advantage of another property of DNA—its programmability. By encoding binary data into DNA origami and reading it as spatially and temporally distinct hybridization events, dNAM decouples information recovery from sequencing. Editing the data is trivial through the inclusion or exclusion of sequence extensions from a library of staple strands. Data strands can be stored directly or incorporated into origami and then stored; separating the 3D storage density from the 2D reading density. In addition, dNAM is a massively parallel process because the large optical field of view affords tens of thousands of origami to be imaged simultaneously, and the number of optical read heads is proportional to the concentration of the imager strands in solution. Rather than averaging thousands of DNA-PAINT images together to resolve the digital data^17^, individual origami were read here using custom encoding, decoding, and error-correction algorithms. Our algorithms combined fountain codes with bi-level parity codes to significantly enhance our data retention—creating a multi-layer error correction scheme that encoded index, orientation, parity, and checksum bits into the origami. As a proof of concept, several bytes of data were recovered in a single DNA-PAINT recording. Even when the DNA origami recovery rate was poor (as low as 63%), the message was recovered 100% of the time. As an alternative platform for testing DNA-memory technology, dNAM offers a pathway to explore the advantages and disadvantages of DNA as a material for information storage and encryption, as previously demonstrated by Zhang et al.^29^. Because of the scaling challenges of using DNA as a memory material, this is particularly true for applications like barcoding that require a limited amount of data to have high information density, redundancy, and copy number.

## Methods

The materials purchased for this study, and their respective vendors, are outlined in Table 1. All other reagents were obtained from Sigma.

**Table 1:** Materials.

Materials purchased	Vendor
DNA staple strands	Integrated DNA Technologies
M13 bacteriophage single-stranded DNA scaffolds (M13mp18)	Bayou Biolabs
Cy3B-labeled DNA oligonucleotide (M1 Imager strand: CTAGATGTAT-Cy3B)	Bio-Synthesis, Inc.
150 nm diameter silanized gold nanoparticles (AuNPs)	Nanopartz
Glass coverslips	Ted Pella, Inc.
Sticky-slide flow cells (sticky-Slide I 0.2 Luer)	Ibidi
Liquinox	Pollardwater, Inc.
MilliporeSigma	MilliporeSigma
Protocatechuate 3,4-dioxygenase pseudomonas (PCD)	MilliporeSigma
(+−)−6-hydroxy-2,5,7,8-tetra-methylchromane-2-carboxylic acid (Trolox)	MilliporeSigma
MgCl_2_	MilliporeSigma
Nuclease-free water	Thermo Fisher Scientific
Tris-borate-EDTA (TBE)	Thermo Fisher Scientific
Tris-Acetate-EDTA (TAE)	Thermo Fisher Scientific

List of materials and vendors used in this study.

### Buffers

As previously described^17^, two buffers were used to prepare and image DNA origami: a deposition buffer and an imaging buffer. The deposition buffer contained 0.5× TBE and 18 mM MgCl_2_. The imaging buffer contained the deposition buffer with the supplement of 60 nM PCD, 1 mM Trolox, 3 nM imager strands, and 10 mM PCA. PCA was added to the imaging buffer immediately before the start of a DNA-PAINT recording.

### Encoding algorithm

The encoding algorithm used a multi-layer error correction scheme to encode message data bits along with the index, orientation, and error correction bits onto multiple origami (Fig. S4).

At the message level, the algorithm used a fountain code to encode the data. Let *m* be a message string composed of a sequence of *n* bits. The fountain code algorithm first divides *m* into *k* equally sized and non-overlapping substrings *s*_*1*_, *s*_*2*_, …, *s*_*k*_, where the concatenation *s*_*1*_*s*_*2*_…*s*_*k*_ = *m*, and then systematically combines one to many segments using the binary XOR operation to form multiple data blocks called droplets. The number of segments d used to form each droplet are typically drawn from a distribution based on the Soliton distribution:

1 $$p\left( 1 \right) = 1/k$$

The Soliton distribution ensures that the algorithm encodes the optimal number of single-segment droplets necessary for the decode step. Once the number of segments *d* for a droplet is determined, the droplet is formed by XOR’ing *d* randomly selected, unique segments from *m*, with each segment being selected with probability 1/*k*.

For our experiments, we divided the message ‘Data is in our DNA!\n’ into 10 segments of 16 bits each. The segments were then combined via an XOR in different combinations using the fountain code algorithm to form the 15 droplets. While the theoretical minimum number of 16-bit droplets required to decode the message is 10, the redundancy provided by the additional droplets ensured that the message would be recoverable in all cases involving the loss of one droplet, and in some cases with the loss of up to five droplets (Fig. 4).

After generating the droplets using fountain codes, the encoding algorithm encoded each droplet onto fifteen 6 × 8 matrixes, and sequentially added index and orientation marker bits, computed and added checksum bits, and then added parity bits (Fig. 1b). These matrixes were used to construct 15 origami structures, with a one-to-one mapping between the matrixes and the origami’s data domains.

Figure 1b shows the layout of how droplet information was encoded onto each origami, composed of 16 bits of droplet data (green coloring in Fig. 1b), four indexing bits (red), four orientation bits (magenta), four checksum bits (yellow), and twenty parity bits (blue). It is important to note that the layout of the data, orientation, and index bits relative to the corresponding parity and checksum bits is invariant to rotation, which made it possible for the error correction algorithm to perform error detection and recovery before determining the orientation (Fig. S4). This led to more robust data recovery.

### DNA origami folding

Rectangular DNA origami structures (~90 × 70 nm) were designed based on previous work by Rafat et al.^30^ with 48 potential docking strand sites arranged in a 6 × 8 matrix with 10 nm spacing. Then, using the protocol described by Schnitzbauer et al.^17^ a mixture of extended and unmodified staple strands (SI Tables S1 and S2) were selected to fold the M13 scaffold into the designed shape, with extended strands located at the ‘1’ positions described in the design matrix (SI Table S4). As described in the introduction, an extended staple strand has a binding site for the M1 imager strand, unmodified strands bind solely to the scaffold DNA to induce folding. Using this method, 15 origami designs were created that matched the 15 matrixes output by the encoding algorithm.

We assembled individual origami designs by combining 22 nM M13mp18 with 10× unmodified stands, 50× extended strands, 1× TAE and 18 mM MgCl_2_ (in nuclease-free water; 100 µL total volume) and folding in a Mastercycler nexus thermal cycler (Eppendorf) using the following heating cycle: [1 min 90 °C, 2 min 80 °C, then from 80 °C to 25 °C over 12 h]. We purified the origami by running them on an ice-cooled 0.8% agarose gel containing 0.5× TBE and 8 mM MgCl_2_, excising the single sharp band, and collecting the exudate of the crushed gel piece. Sharp triangle origami used as fiducial markers were prepared similarly, as previously described^31^ (see S1 Table S3 for oligonucleotide sequences). All purified origami were stored in the dark at 4 °C until use.

### Glass coverslip preparation

Borosilicate glass coverslips (25 × 75 and 22 × 22 mm, #1 Gold Seal Coverglass) were sonicated in 0.1% (v/v) Liquinox and nano-pure water (1 min in each) to remove contaminants and dried at 40 °C for at least 30 min. Fiducial markers (200 µL of 0.2 pM AuNPs) were deposited onto the coverslips for 10 min at room temperature. The labeled coverslips were rinsed with methanol and nano-pure water and stored at 40 °C prior to use.

### DNA origami deposition onto coverslips

The glow discharge technique previously described by Green^24^ was used to deposit DNA origami onto glass coverslips using an air-plasma vacuum glow-discharge system. Briefly, coverslips that had been cleaned and labeled with fiducial markers were exposed to glow discharge generated using an electrode coupled 115 V Electro-Technic BD-10A High-Frequency Generator under 2 Torr of vacuum for 75 s. For DNA-PAINT analysis, a sticky-Slide flow cell (~50 µL channel volume) was glued to the coverslip, DNA origami were then deposited by introducing 200 µL of 0.05 nM origami (a mixture of dNAM origami and sharp triangle origami^31^ added as additional fiducial markers, in deposition buffer) into the flow chamber and incubated for 30 min at room temperature. After deposition, the flow chamber was rinsed with 1 mL of deposition buffer (no DNA origami) and refilled with imaging buffer.

When performing AFM measurements on samples previously used for DNA-PAINT, a custom fluid chamber, modified from Jungmann et al.^32^, was used. A 22 × 22 mm coverslip was glued to a microscope slide using double-sided sticky tape with the addition of a thin layer of gel sealant—to both seal any gaps and weaken the binding of tape to the glass. Once DNA-PAINT imaging had been performed the sealant allowed the coverslip to be easily removed for further AFM analysis.

### Fluorescence microscopy

DNA origami was imaged below the diffraction limit of light via DNA-PAINT^17^ using an inverted Nikon Eclipse Ti2 microscope from Nikon Instruments in total internal reflectance fluorescence (TIRF) mode. The images were acquired using: an optical feedback focal-drift correction system developed in-house or the Perfect Focus System from Nikon Instruments; an oil-immersion CFI Apochromat ×100 TIRF objective with a 1.49 numerical aperture, plus an extra ×1.5 magnification from Nikon Instruments; and a 405/488/561/647 nm Laser Quad Band Set TIRF filter cube from Chroma. A 561 nm laser source excited fluorescence from the DNA-PAINT imager strands within an evanescent field extending a few hundred nanometers above the surface of the glass coverslip. The emitted fluorescence was imaged onto the full chip with 512 × 512 pixels (1 pixel = 16 μm) using a ProEM EMCCD camera from Princeton Instruments at a 300 ms exposure time (~3 frames/s). During an experimental recording, each of the individual data strands, within a dNAM origami’s matrix, transiently and repeatedly bound an imager strand, which emits a signal, creating a series of blinks. Images with blinking events were recorded into a stack (typically 40,000 frames per recording) using Nikon NIS-Elements version 5.20.00 (Nikon Instruments) or LightField version 5 (Princeton Instruments) prior to processing and analysis.

### DNA-PAINT fluorophore localization

After recording a DNA-PAINT stack, the center position of signals (localizations) emitted by imager probes, transiently binding to DNA origami docking strands, were identified using the ImageJ ThunderSTORM plugin^33^. The localizations were rendered and then drift corrected using the Picasso-Render software package, as described by Schnitzbauer et al.^17^. Data visualization and peak fitting of image data for PSF analysis were performed using OriginPro Version 2019b (OriginLab).

### Localization data processing

A custom algorithm was developed for identifying clusters of localizations, determining the maximum likelihood position of the emitters, and generating binary matrix data. The algorithm selected localization clusters at random from the localization list. To do this, it sampled random points in the list, determined the average position of nearby localizations, and counted the localizations within a radius (*R*) and the localizations within a band *R* < *r* < 2*R*. The algorithm accepted clusters if the counts in the inner circle were greater than a threshold and the counts in the outer band were less than 15% of the counts in the inner band. This ensured selection of bright clusters that were isolated from other clusters.

The algorithm then fits the cluster localizations to a grid of emitters. An idealized grid was created using the average DNA-PAINT image produced by several thousand individual origami structures of the same architecture used in this work. The algorithm performed fitting using a maximum likelihood estimation for the likelihood function:

2 $$L\left( {I,x_c,y_c,\theta ,{\mathrm{{\Delta}}}x_g^2,B} \right) = \mathop {\prod }\limits_i \left( {\mathop {\sum }\limits_k \frac{{I_k}}{a}\exp \left( { - \frac{{\left( {x_i - x_k\left( {x_c,y_c,\theta } \right)} \right)^2 + \left( {y_i - y_k\left( {x_c,y_c,\theta } \right)} \right)^2}}{{{\mathrm{{\Delta}}}x_i^2 + {\mathrm{{\Delta}}}x_g^2}}} \right)} \right) \ast \frac{B}{A} \ast P(N,I,B)$$

Where *I*_*k*_ is the intensity of the *k*^th^ emitter, (*x*_*c*_, *y*_*c*_) is the center position of the grid, *θ* is the rotation angle of the grid, Δ*x*_*g*_ is the global lateral uncertainty caused by an error in drift correction, *B* is the background, Δ*x*_*i*_ is the lateral position uncertainty of localization *i* reported by the ThunderSTORM analysis described above, (*x*_*i*_, *y*_*i*_) is the position of the *i*th localization, (*x*_*k*_, *y*_*k*_) is the position of the *k*^th^ emitter, as a function of the center position and rotation of the grid, *A* is the area of the cluster, and *N* is the number of localizations found in the cluster. *a* is a normalization constant given by:

3 $$a = 2\pi \left( {{\mathrm{{\Delta}}}x_i^2 + {\mathrm{{\Delta}}}x_g^2} \right)$$

*P(N,I,B)* is the probability of finding *N* localizations given the intensity of each grid point and the background intensity, determined from the Poisson distribution of mean value *N*. This likelihood function determines the probability of finding localizations at all of the observed sites given a set of point emitters at the grid sites with intensity *I*_*k*_ and background intensity *B*. The optimization utilized the L-BFGS-B method of the minimize function provided by Scipy^34^ to minimize −log*(L)* subject to the constraint that all intensities are positive. Signals that did not align to the 6 × 8 grid were filtered to minimize fragmented origami and to reduce inadvertent assimilation of the triangular origami fiducial markers into the results.

The algorithm then assigned the emitters a binary value (1 or 0) using an empirically derived threshold value. This binary matrix data was decoded using the decoding algorithm described below.

In parallel with this blind cluster analysis, the processing algorithm also carried out a template matching step to more reliably identify individual origami and analyze their errors. This additional step used the known origami designs as templates, matching the observed origami to the best fit, based on the total number of errors. This method was more robust to higher error rates than the blind cluster analysis and allowed more origami to be identified for image averaging and error analysis (Fig. 3). It should be noted, however, that the template matching method cannot be considered as a data reading method because it requires a priori knowledge of the data being analyzed. For this reason, none of the analysis of the recovery rates or data density discussed here used data obtained from pattern matching.

### Decoding algorithm

The decoding algorithm (Fig. S5) utilized a multi-layer error correction/encoding scheme to recover the data in the presence of errors. The algorithm first works at the dNAM origami level (Step 1, below), using the parity and checksum bits, to attempt to identify and correct errors and recover the correct matrix. After recovery, the algorithm uses binary operations to recover the original data segments from the droplets (Step 2, below).

### Decoding algorithm: Step 1–error correction

Given raw binary matrix data **M** for a single dNAM origami, the output from the localization data processing step, the matrix decoding algorithm determined which, if any, bits were associated with checksum and parity errors by calculating the bi-level matrix parity and checksum values, as described in Fig. S4. Any discrepancies between the calculated parity and checksum values and the values recovered from the origami were noted, and a weight for each of the bits associated with the errant parity/checksum calculation was deduced. If no parity/checksum errors were detected for a particular matrix, then the data was assumed to be accurate, and the algorithm proceeded to extract the message data.

To determine the site(s) of likely errors, the decoding algorithm first determined a weight for every cell in **M**, beginning with data cells (the cells containing droplet, index, or orientation bits) and proceeding to parity and checksum cells. Let $P_{c_{ij}}$ be the set of parity functions calculated over a given data cell *c*_*ij*_. Then for each data cell *c*_*ij*_:

4 $$x_{ij} = \mathop {\sum }\limits_{f_{c_{pq}} \in P_{c_{ij}}} \left| {c_{pq} - f_{c_{pq}}\left( {\mathbf{M}} \right)} \right|$$

Where *c*_*pq*_ is the parity cell where the expected binary value of *f* is stored.

The weight for each parity cell *c*_*ij*_ was then calculated based on the number of non-zero weights greater than 1 for the data cells associated with it. More formally, let *c*_*ij*_ be a parity cell and $D_{c_{ij}}$ be the set of data cells used in the calculation of *c*_*ij*_. Then the weight *x*_*ij*_ for each parity cell *c*_*ij*_ is:

5 $$x_{ij} = \mathop {\sum }\limits_{c_{pq} \in D_{c_{ij} \wedge x_{pq} > 1}} {\mathop{\rm{sgn}}} \left( {x_{pq}} \right)$$

The higher the weight value, the higher the probability that the corresponding cell had an error.

An overall score for the matrix was then calculated by summing over all *x*_*ij*_ and normalizing by the sum of the correctly matched parity bits. This value was designated as the overall weight of the matrix. Higher values of this weight correspond to matrixes with more errors.

6 $${\mathrm{Overall}}\,{\mathrm{matrix}}\,{\mathrm{weight}} = \frac{{\mathop {\sum }\nolimits_{i = 0}^6 \mathop {\sum }\nolimits_{j = 0}^8 x_{ij}}}{{\# {\mathrm{number}}\,{\mathrm{of}}\,{\mathrm{matched}}\,{\mathrm{parity}}\,{\mathrm{bits}}}}$$

The algorithm then performed a greedy search to correct the errors using a priority queue ordered by the overall matrix weight (Fig. S6). The algorithm began by iteratively altering each of the probable site errors and computing the overall matrix weight of the modified matrix for each, placing each potential bit flip into a priority queue where the flips that produced the lowest overall weights had the highest priority. At each step, the algorithm selected the bit flip associated with the highest priority in the queue and then repeated this process on the resulting matrix. This process was continued until the algorithm produced a matrix with no mismatches or until it reached the maximum number of allowed bit flips (9 for our simulation/experiment). If it reached the maximum number of flips, it returned to the queue to pursue the next highest priority path. If the algorithm found a matrix with no mismatches, it then checked the orientation bits and oriented the matrix accordingly. The droplet and index data were then extracted and passed to the next step. If the queue was emptied without finding a correct matrix, the algorithm terminated in failure.

### Decoding algorithm: Step 2–fountain code decoding

After extracting the droplet and index data from multiple origami the algorithm attempted to recover the full message (Fig. S7). Once decoded, each droplet had one or multiple segments XORed in it. Using the recovered indexes the algorithm determined how many and which segments were contained in each droplet. To decode the message, the algorithm maintained a priority queue of droplets based on the number of segments they contained (their degree), with the lowest degree droplets having the highest priority. The algorithm looped through the queue, removing the lowest degree droplet, attempting to use it to reduce the degree of the remaining droplets using XOR operations, and re-queuing the resulting droplets. Upon finding a droplet of ‘degree one’ it stored it as a segment for the final message. If all segments were recovered, the algorithm terminated successfully.

### Data simulation test

To test the robustness of our encoding and decoding algorithms, origami data were simulated with randomly generated messages and errors. First, random binary messages of size *m* were created (for *m* = 160 to 12,800 bits, at 320-bit intervals). These messages were then divided into *m/b* equally sized segments, where *b* is the number of data bits to be encoded onto an individual origami. For fixed-size origami, larger messages necessitated a smaller *b*, as more bits had to be dedicated to the index. In these cases, *b* varied between eight (for *m* = 12,800) and twelve (for *m* = 160). After determining message segments, droplets were formed using the fountain code algorithm and encoded onto origami, along with the corresponding index, orientation, and error-correcting bits. Ten in silico copies of each unique origami were created, and 0–9 bits flipped at random to introduce errors. The origami was decoded as described above.

### Reporting summary

Further information on research design is available in the Nature Research Reporting Summary linked to this article.

## Supplementary information

Supplementary Information

Peer Review File

Reporting Summary

## Data Availability

DNA-PAINT images were analyzed using custom and publicly available codes (as indicated). The encoding/decoding algorithms were written in-house using Python, version 3.7.3. The source codes for the encoding, decoding, and localization algorithms are available on GitHub at https://github.com/BoiseState/NAM. The schematic in Fig. 1c of digital Nucleic Acid Memory was derived from a model created using Nanodesign (www.autodeskresearch.com/projects/nanodesign).

## Source data

Source Data

## Data availability

The original DNA-PAINT recordings and drift-corrected centroid localization data that support the findings of this study have been deposited in the Zenodo repository with the identifier “10.5281/zenodo.4672665”. Source data are provided with this paper. Any other relevant data are available from the authors upon reasonable request. Source data are provided with this paper.
