# Supplementary material for: An alternative approach to nucleic acid memory
Source: Nat Commun. 2021 Apr 22;12:2371. doi: 10.1038/s41467-021-22277-y (PMC8062470; doi:10.1038/s41467-021-22277-y)
Supplement: Supplementary file 1 — Supplementary Information [file 41467_2021_22277_MOESM1_ESM.pdf]

Supplementary Information – An Alternative Approach to Nucleic Acid Memory

George D. Dickinson[a±], Golam Md Mortuza[b±], William Clay[a±], Luca Piantanida[a±], Christopher M. Green[a,c], Chad Watson[a], Eric J. Hayden[d], Tim Andersen[b], Wan Kuang[e], Elton Graugnard[a], Reza Zadegan[a,f] and William L. Hughes[a*]

[a] Micron School of Materials Science & Engineering, Boise State University, Boise, ID 83725, USA.

[b] Department of Computer Science, Boise State University, Boise, ID 83725, USA.

[c] Now at the Center for Bio/Molecular Science & Engineering, U.S. Naval Research Laboratory, Washington, D.C. 20375, USA.

[d] Department of Biological Sciences, Boise State University, Boise, ID 83725, USA.

[e] Department of Electrical & Computer Engineering, Boise State University, Boise, ID 83725, USA.

[f] Now at the Department of Nanoengineering, Joint School of Nanoscience and Nanoengineering, North Carolina A&T State University, Greensboro, NC 27410, USA.

± Contributed equally

* Corresponding author

Table of Contents

Glossary of Terms *3*

Supplementary Materials and Methods *6*

Oligonucleotide Sequences 6

Encoding/Decoding Algorithms 19

Atomic Force Microscopy 19

Supplementary Results *20*

DNA-PAINT Resolution 20

Proximity Error Analysis 21

Supplementary Diagrams and Charts *23*

Figure S4. The Encoding Algorithm 23
Figure S5. The Decoding Algorithm 28
Figure S6. Flowchart 1 - Error Correction 29
Figure S7. Flowchart 2 - Fountain Code Decoding 30
Figure S8. dNAM Summary 31

Supplementary Notes *32*

Writing Speed 32

1. Echo liquid-handling system capacity 32
Calculation 32
2. TWIST Bioscience DNA synthesis 32
Calculation 32
3. Magnetic Tape 32

Storage—Data Density 33

1. dNAM, unfolded data strands 33
Calculations 33
2. dNAM, data strands folded into origami 33
Calculations 33
1. DNA Storage capacity 34
2. Magnetic Tape Capacity 34
Calculation 34

Reading Speeds 35

1. dNAM/DNA-PAINT 35
Calculation 35
2. NovaSeq 6000 sequencer 35
3. Magnetic Tape 35

Supplementary References *37*

Glossary of Terms

AFM: Atomic Force Microscopy, scanning probe microscopy with a resolution < 1 nm. Uses a cantilever and a probe to physically scan the surface of a sample.

Archival storage: Storage of inactive data. Typically, data that is rarely accessed but needs to be retained for long periods of time.

Binary string: Here a sequence of bits (i.e. a sequence of 0's and 1's). It can also be used to describe a sequence of bytes – for example, for an 8-bit byte a sequence in which every element is 8-bits long.

Bit: A binary digit, the smallest unit of information used by a computer. In dNAM, a bit is encoded by the data strand.

Byte: The smallest addressable unit of memory used by a computer, made up of bits (typically 8) and originally used to encode a single character of text.

Checksum bit: A bit of the matrix which contains the checksum value from a subset of data bits, orientation bits, and indexing bits.

Data bit: A bit of the matrix which contains a bit of information from XORed segments of the message being encoded.

Data strand: Selected staple strands within a dNAM origami that are used to encode information, as described in the text. Data strands representing a zero (0) consist of only the staple strand domain. Data strands representing a one (1) consist of the staple strand domain extended by a docking domain, which acts as a docking site for complementary data *imager* strands. Data strands are the information bearing particles in the origami, analogous to the magnetic particles coating a tape or disk used in a tape recorder or hard drive for magnetic recording.

Decoding algorithm: The algorithm used to decode messages from individual matrixes.

Degree distribution: The distribution of the segments into the droplet.

dNAM: Digital NAM, a type of NAM in which information is encoded into defined spatial arrangements of DNA sequences on top of addressable DNA origami nanostructures.

dNAM origami: A single rectangular 2D DNA origami nanostructure with specific sequences used to localize data strands to specific sites on the upper surface. This site-specific localization is enabled by extending (1) or not extending (0) the structural staple strands of the DNA origami to create addressable data strands.

DNA origami: A self-assembling nanostructure formed through the interactions of numerous short single-strands of DNA (staples) with a long single-strand of M13 DNA (scaffold). The scaffold is folded during assembly, bringing together B-form double helices with their helical axes parallel to each other, and the structure is held together by crossovers between neighboring helices.

DNA-PAINT: DNA-Points Accumulation for Imaging in Nanoscale Topography, a DNA based form of stochastic SRM that uses repetitive, transient binding of fluorescently labelled imager strands to circumvent the diffraction limit of light.

Droplet: A chunk of data created by a fountain code during transmission of a larger message.

Greedy algorithm: A type of algorithm that attempts to determine a globally-optimal solution to a problem by making locally-optimal choices at each search step. It uses a heuristic to determine each choice, such as: always choose the smallest, largest, etc.

Imager strand: A short, fluorescently labelled, single strand of DNA, complementary to the docking domain of a data strand that encodes a one (1) in a dNAM origami. In dNAM, imager strands act as the read head and reveal the location of the ones in the dNAM origami.

Index bit: A bit of the matrix that is used to encode a unique identifier for each droplet that allows the algorithm to determine the exact message segments that are encoded in the matrix.

Matrix: In dNAM, the 2-dimensional representation of the binary data, index, orientation marker, parity, and checksum bits encoded on the DNA.

NAM: Nucleic Acid Memory, a memory-storage material comprised of nucleic acids that has the potential for high volumetric density, long retention times, and low energy of operation.

Orientation bit: A bit of the matrix which indicates the orientation of the matrix.

Packet: A unit of data made into a single package for transmission over a digital network.

Parity bit: A bit of the matrix which contains the XORed value from a subset of data bits, orientation bits, indexing bits, and checksum bits, providing a second level of error correction capability.

Matrix weight: A float value calculated using the parity and checksum bits that indicates the presence of an error in the matrix.

Priority queue: A queue data type with each element in the queue has a priority value assigned. Abbreviated to pqueue here. Elements with high priority are served before elements with low priority.

Read head: The component of a recording device that senses the information stored in a memory material. Typically, an electromechanical mechanism that converts the magnetic field of a section of tape or disk platter into an electrical current. In dNAM the imager strands act as read heads.

Scaffold DNA: A long single-stranded DNA oligonucleotide (here, a 7249-nucleotide long loop of M13 phage DNA) that is folded via the binding of hundreds of shorter DNA oligonucleotides to form a self-assembling nanostructure. The shape of the resulting DNA-origami is determined by the choice of staples and scaffold (which runs throughout the structure) in a highly predictable manner - allowing rational design of complex 2 and 3-D structures.

SRM: Super-Resolution Microscopy, a class of light microscope techniques capable of surpassing the optical diffraction resolution limit of ~250 nm.

Staple strand: A short (typically 15-60 nucleotides) single-stranded DNA oligonucleotide used to fold scaffold DNA during the formation of DNA origami. The staple strands form crossovers between neighboring scaffold strand helices.

XOR operation: The binary exclusive OR operation (⊕) in which corresponding bits of a binary number are compared and yields true (1) if exactly one of two conditions is true (false = 0), see Table 1. For multiple arguments, XOR is defined to be true if an odd number of its arguments are true, and false otherwise (equivalent to addition modulo 2). See Table 2 for a three-argument function.

Table 1

a	b	$a \oplus b$
0	0	0
0	1	1
1	0	1
1	1	0

Table 2

a	b	c	$a \oplus b \oplus c$
1	1	1	1
1	1	0	0
1	0	1	0
1	0	0	1
0	1	1	0
0	1	0	1
0	0	1	1
0	0	0	0

'\n' character: In many programming languages '\n' represents a newline escape character (i.e. an instruction to insert a newline at this point).

Supplementary Materials and Methods

Oligonucleotide Sequences

Name	Sequence	Role
n0-Ptt-1LR2-1LR1-Ptt	TTTTCACGTTGAAAATCTCGCGAATAATAATTTTTTTT	Registration
n0-Ptt-1LR4-1LR3-Ptt	TTTAGGAAGTTTCCATTAATAAAGACTTTTTCATGTTT	Registration
n0-Ptt-1LR6-1LR5-Ptt	TTTCAGGCGCATAGGCTGGTGAACGGTGTACAGACTTT	Registration
n0-Ptt-1LR8-1LR7-Ptt	TTTGGTAGAAAGATTCATCGAACAACATTATTACATTT	Registration
n0-Ptt-1LR10-1LR9-Ptt	TTTTGACCATAAATCAAAAGTTCAGAAAACGAGAATTT	Registration
n0-Ptt-1LR12-1LR11-Ptt	TTTGTGTCTGGAAGTTTCAATGCAACTAAAGTACGTTT	Registration
n0-Ptt-1LR14-1LR13-Ptt	TTTTTTTGCGGGAGAAGCCTATGACCCTGTAATACTTT	Registration
n0-Ptt-1LR16-1LR15-Ptt	TTTGTCAATCATATGTACCATCGTAAAACTAGCATTTT	Registration
n0-Ptt-1LR18-1LR17-Ptt	TTTGTGTAGATGGGCGCATGGGATAGGTCACGTTGTTT	Registration
n0-Ptt-1LR20-1LR19-Ptt	TTTAGTGCCAAGCTTGCATTTGTAAAACGACGGCCTTT	Registration
n0-Ptt-1LR22-1LR21-Ptt	TTTTATTGGGCGCCAGGGTGGAGAGGCGGTTTGCGTTT	Registration
n0-Ptt-1LR24-1LR23-Ptt	TTTTGGCCCACTACGTGAACCGTCTATCAGGGCGATTT	Registration
n0-Ptt-RR1-RR2-Ptt	TTTCAGAACCGCCACCCTCTCAGAACCGCCACCCTTTT	Registration
n0-Ptt-RR3-RR4-Ptt	TTTATACAGGAGTGTACTGTACATGGCTTTTGATGTTT	Registration
n0-Ptt-RR5-RR6-Ptt	TTTCGTTTGCCATCTTTTCATAGCCCCCTTATTAGTTT	Registration
n0-Ptt-RR7-RR8-Ptt	TTTCAAAGACAAAAGGGCGTATGGTTTACCAGCGCTTT	Registration
n0-Ptt-RR9-RR10-Ptt	TTTAGAGCAAGAAACAATGGTTAAGCCCAATAATATTT	Registration
n0-Ptt-RR11-RR12-Ptt	TTTCAATTTTATCCTGAATATTTTGCACCCAGCTATTT	Registration
n0-Ptt-RR13-RR14-Ptt	TTTTATCCCATCCTAATTTTGAACAAGAAAAATAATTT	Registration
n0-Ptt-RR15-RR16-Ptt	TTTCATAATTACTAGAAAAGAATAAACACCGGAATTTT	Registration
n0-Ptt-RR17-RR18-Ptt	TTTAATCCTTGAAAACATAATTAATTTTCCCTTAGTTT	Registration
n0-Ptt-RR19-RR20-Ptt	TTTAGATGAATATACAGTATTTCAGGTTTAACGTCTTT	Registration
n0-Ptt-RR21-RR22-Ptt	TTTAGACTTTACAAACAATAGGATTTAGAAGTATTTTT	Registration
n0-Ptt-RR23-RR24-Ptt	TTTAAAAATACCGAACGAACTAAAACATCGCCATTTTT	Registration
n0--A8-A10-	TTTAGGACAAATGCTTTAAACAATCAGGTC	Blocking
n0--A10-A12-	TTTACCCCAACATGTTTTAAATTTCCATAT	Blocking
n0--A12-A14-	AACAGTTTTGTACCAAAAACATTTTATTTC	Blocking
n0--A14-A16-	AACGCAAAATCGATGAACGGTACCGGTTGA	Blocking
n0--A16-A18-	TAATCAGCGGATTGACCGTAATCGTAACCG	Blocking
n0--B1-A2-	AGAAAGGAACAACTAAAGGAATTCAAAAAAA	Blocking
n0--A18-A20-	TGCATCTTTCCCAGTCACGACGGCCTGCAG	Blocking

n0--A20-A22-	GTCGACTTCGGCCAACGCGCGGGGTTTTTC	Blocking
n0--A22-A24-	TTTTCACTCAAAGGGCGAAAAACCATCACC	Blocking
n0--A2-A4-	AGGCTCCAGAGGCTTTGAGGACACGGGTAA	Blocking
n0--A4-A6-	AATACGTTTGAAAGAGGACAGACTGACCTT	Blocking
n0--A6-A8-	CATCAAGTAAAACGAACTAACGAGTTGAGA	Blocking
n0--B3-B1-	ACGGCTACAAAAGGAGCCTTTAATGTGAGAAT	Blocking
n0--B13-B11-	TAAATCGGGATTCCCAATTCTGCGATATAATG	Blocking
n0--B15-B13-	AACAAGAGGGATAAAAATTTTTAGCATAAAGC	Blocking
n0--B17-B15-	ACAAACGGAAAAGCCCCAAAAACACTGGAGCA	Blocking
n0--B19-B17-	CCAGGGTTGCCAGTTTGAGGGGACCCGTGGGA	Blocking
n0--B21-B19-	TTAATGAACTAGAGGATCCCCGGGGGGTAACG	Blocking
n0--B23-B21-	CTCCAACGCAGTGAGACGGGCAACCAGCTGCA	Blocking
n0--A24-B23-	CAAATCAAGTTTTTTGGGGTCGAAACGTGGA	Blocking
n0--B5-B3-	GACCAACTAATGCCACTACGAAGGGGGTAGCA	Blocking
n0--B7-B5-	TACGTTAAAGTAATCTTGACAAGAACCGAACT	Blocking
n0--B9-B7-	ATCCCCCTATACCACATTCAACTAGAAAAATC	Blocking
n0--B11-B9-	CTGTAGCTTGACTATTATAGTCAGTTCATTGA	Blocking
n0--C8-C10-	ACATAACGGGAATCGTCATAAATAAAGCAAAG	Blocking
n0--C10-C12-	CGGATTGCAGAGCTTAATTGCTGAAACGAGTA	Blocking
n0--C12-C14-	GATTTAGTCAATAAAGCCTCAGAGAACCCTCA	Blocking
n0--C14-C16-	TATATTTTGTCATTGCCTGAGAGTGGAAGATT	Blocking
n0--C16-C18-	GTATAAGCCAACCCGTCGGATTCTGACGACAG	Blocking
n0--D1-C2-	ACAACTTTCAACAGTTTCAGCGGATGTATCGG	Blocking
n0--C18-C20-	TATCGGCCGCAAGGCGATTAAGTTTACCGAGC	Blocking
n0--C20-C22-	TCGAATTCGGGAAACCTGTCGTGCAGCTGATT	Blocking
n0--C22-C24-	GCCCTTCAGAGTCCACTATTAAAGGGTGCCGT	Blocking
n0--C2-C4-	TTTATCAGGACAGCATCGGAACGACACCAACC	Blocking
n0--C4-C6-	TAAAACGAGGTCAATCATAAGGGAACCGGATA	Blocking
n0--C6-C8-	TTCATTACGTCAGGACGTTGGGAAATGCAGAT	Blocking
n0--D3-D1-	CAGCGAAACTTGCTTTCGAGGTGTTGCTAA	Blocking
n0--D13-D11-	AAATTAAGTTGACCATTAGATACTTTTGCG	Blocking
n0--D15-D13-	GCTATCAGAAATGCAATGCCTGAATTAGCA	Blocking
n0--D17-D15-	GCGAGTAAAAATATTTAAATTGTTACAAAG	Blocking
n0--D19-D17-	GATGTGCTTCAGGAAGATCGCACAATGTGA	Blocking
n0--D21-D19-	TTCCAGTCGTAATCATGGTCATAAAAGGGG	Blocking
n0--D23-D21-	TGGAACAACCGCCTGGCCCTGAGGCCCGCT	Blocking
n0--C24-D23-	AAAGCACTAAATCGGAACCCTAATCCAGTT	Blocking
n0--D5-D3-	GCGCAGACAAGAGGCAAAAGAATCCCTCAG	Blocking
n0--D7-D5-	TTATACCACCAAATCAACGTAACGAACGAG	Blocking
n0--D9-D7-	AATACTGCCCAAAAGGAATTACGTGGCTCA	Blocking
n0--D11-D9-	GATGGCTTATCAAAAAGATTAAGAGCGTCC	Blocking

n0--E8-E10-	TAAGAGCAAATGTTTAGACTGGATAGGAAGCC	Blocking
n0--E10-E12-	CGAAAGACTTTGATAAGAGGTCATATTTCGCA	Blocking
n0--E12-E14-	AATGGTCAACAGGCAAGGCAAAGAGTAATGTG	Blocking
n0--E14-E16-	TAGGTAAACTATTTTTGAGAGATCAAACGTTA	Blocking
n0--E16-E18-	ATATTTTGGCTTTCATCAACATTATCCAGCCA	Blocking
n0--F1-E2-	TAAATGAATTTTCTGTATGGGATTAATTTCTT	Blocking
n0--E18-E20-	GCTTTCCGATTACGCCAGCTGGCGGCTGTTTC	Blocking
n0--E20-E22-	CTGTGTGATTGCGTTGCGCTCACTAGAGTTGC	Blocking
n0--E22-E24-	AGCAAGCGTAGGGTTGAGTGTTGTAGGGAGCC	Blocking
n0--E2-E4-	AAACAGCTTTTTGCGGGATCGTCAACACTAAA	Blocking
n0--E4-E6-	ACACTCATCCATGTTACTTAGCCGAAAGCTGC	Blocking
n0--E6-E8-	TCATTCAGATGCGATTTTAAGAACAGGCATAG	Blocking
n0--F3-F1-	AAGGCCGCTGATACCGATAGTTGCGACGTTAG	Blocking
n0--F13-F11-	TAAATCATATAACCTGTTTAGCTAACCTTTAA	Blocking
n0--F15-F13-	GAGGGTAGGATTCAAAAGGGTGAGACATCCAA	Blocking
n0--F17-F15-	TGTAGCCATTAAAATTCGCATTAAATGCCGGA	Blocking
n0--F19-F17-	TCTTCGCTGCACCGCTTCTGGTGCGGCCTTCC	Blocking
n0--F21-F19-	CACATTAAAATTGTTATCCGCTCATGCGGGCC	Blocking
n0--F23-F21-	GCCCGAGAGTCCACGCTGGTTTGCAGCTAACT	Blocking
n0--E24-F23-	CCCGATTTAGAGCTTGACGGGGAAAAAGAATA	Blocking
n0--F5-F3-	GACCTGCTCTTTGACCCCCAGCGAGGGAGTTA	Blocking
n0--F7-F5-	ATTACCTTTGAATAAGGCTTGCCCAAATCCGC	Blocking
n0--F9-F7-	AATAGTAAACACTATCATAACCCTCATTGTGA	Blocking
n0--F11-F9-	TTGCTCCTTTCAAATATCGCGTTTGAGGGGGT	Blocking
n0--G8-G10-	AGACGACAAAGAAGTTTTGCCATAATTCGA	Blocking
n0--G10-G12-	GCTTCAATCAGGATTAGAGAGTTATTTTCA	Blocking
n0--G12-G14-	TTTGGGGATAGTAGTAGCATTAAAAGGCCG	Blocking
n0--G14-G16-	GAGACAGCTAGCTGATAAATTAATTTTTGT	Blocking
n0--G16-G18-	TAAATCAAAATAATTCGCGTCTCGGAAACC	Blocking
n0--H1-G2-	TCTAAAGTTTTGTCGTCTTTCCAGCCGACAA	Blocking
n0--G18-G20-	AGGCAAAGGGAAGGGCGATCGGCAATTCCA	Blocking
n0--G20-G22-	CACAACAGGTGCCTAATGAGTGCCCAGCAG	Blocking
n0--G22-G24-	GCGAAAAATCCCTTATAAATCAAGCCGGCG	Blocking
n0--G2-G4-	TGACAACTCGCTGAGGCTTGCATTATACCA	Blocking
n0--G4-G6-	AGCGCGATGATAAATTGTGTCGTGACGAGA	Blocking
n0--G6-G8-	AACACCAAATTTCAACTTTAATCGTTTACC	Blocking
n0--J1-H1-	TCCACAGACAGCCCTCATAGTTAGCGTAACGA	Blocking
n0--I10-H11-	AGAGAGAAAAAAATGAAAATAGCAAGCAAACT	Blocking
n0--I12-H13-	CCAATAGCTCATCGTAGGAATCATGGCATCAA	Blocking
n0--I14-H15-	GTAATAAGTTAGGCAGAGGCATTTATGATATT	Blocking
n0--I16-H17-	ATCGCAAGTATGTAAATGCTGATGATAGGAAC	Blocking

n0--I18-H19-	AGAAAACAAAGAAGATGATGAAACAGGCTGCG	Blocking
n0--I20-H21-	GCAATTCACATATTCCTGATTATCAAAGTGTA	Blocking
n0--I22-H23-	TCAATATCGAACCTCAAATATCAATTCCGAAA	Blocking
n0--I2-H3-	TTAGGATTGGCTGAGACTCCTCAATAACCGAT	Blocking
n0--I4-H5-	TTGACAGGCCACCACCAGAGCCGCGATTTGTA	Blocking
n0--I6-H7-	GCAAGGCCTCACCAGTAGCACCATGGGCTTGA	Blocking
n0--I8-H9-	TTATTACGAAGAACTGGCATGATTGCGAGAGG	Blocking
n0--H11-I10-	CCAACAGGAGCGAACCAGACCGGAGCCTTTAC	Blocking
n0--H13-I12-	TTCTACTACGCGAGCTGAAAAGGTTACCGCGC	Blocking
n0--H15-I14-	CAACCGTTTCAAATCACCATCAATTCGAGCCA	Blocking
n0--H17-I16-	GCCATCAAGCTCATTTTTTAACCACAAATCCA	Blocking
n0--H19-I18-	CAACTGTTGCGCCATTCGCCATTCAAACATCA	Blocking
n0--H3-I2-	ATATTCGGAACCATCGCCCACGCAGAGAAGGA	Blocking
n0--H21-I20-	AAGCCTGGTACGAGCCGGAAGCATAGATGATG	Blocking
n0--H23-I22-	TCGGCAAATCCTGTTTGATGGTGGACCCTCAA	Blocking
n0--G24-I24-	AACGTGGCGAGAAAGGAAGGGAAACCAGTAA	Blocking
n0--H5-I4-	TCATCGCCAACAAAGTACAACGGACGCCAGCA	Blocking
n0--H7-I6-	GATGGTTTGAACGAGTAGTAAATTTACCATTA	Blocking
n0--H9-I8-	CTTTTGCAGATAAAAACCAAAATAAAGACTCC	Blocking
n0--J3-J1-	TATTAAGAAGCGGGGTTTTGCTCGTAGCAT	Blocking
n0--J13-J11-	TTTTATTTAAGCAAATCAGATATTTTTTGT	Blocking
n0--J15-J13-	CATGTAATAGAATATAAAGTACCAAGCCGT	Blocking
n0--J17-J15-	TATAACTAACAAAGAACGCGAGAACGCCAA	Blocking
n0--J19-J17-	CTGAGCAAAAATTAATTACATTTTGGGTTA	Blocking
n0--J21-J19-	ATTATCATTCAATATAATCCTGACAATTAC	Blocking
n0--J23-J21-	ACCTTGCTTGGTCAGTTGGCAAAGAGCGGA	Blocking
n0--I24-J23-	TAAAAGGGACATTCTGGCCAACAAAGCATC	Blocking
n0--J5-J3-	CACCAGAAAGGTTGAGGCAGGTCATGAAAG	Blocking
n0--J7-J5-	CAGCAAAAGGAAACGTCACCAATGAGCCGC	Blocking
n0--J9-J7-	ATACCCAACAGTATGTTAGCAAATTAGAGC	Blocking
n0--J11-J9-	TTAACGTCTAACATAAAAACAGGTAACGGA	Blocking
n0--K8-K10-	ATACATACCGAGGAAACGCAATAAGAAGCGCA	Blocking
n0--K10-K12-	TTAGACGGCCAAATAAGAAACGATAGAAGGCT	Blocking
n0--K12-K14-	TATCCGGTCTCATCGAGAACAAGCGACAAAAG	Blocking
n0--K14-K16-	GTAAAGTAATCGCCATATTTAACAAAACTTTT	Blocking
n0--K16-K18-	TCAAATATAACCTCCGGCTTAGGTAACAATTT	Blocking
n0--L1-K2-	TCACCAGTACAAACTACAACGCCTAGTACCAG	Blocking
n0--K18-K20-	CATTTGAAGGCGAATTATTCATTTTTGTTTGG	Blocking
n0--K20-K22-	ATTATACTAAGAAACCACCAGAAGTCAACAGT	Blocking
n0--K22-K24-	TGAAAGGAGCAAATGAAAAATCTAGAGATAGA	Blocking
n0--K2-K4-	GCGGATAACCTATTATTCTGAAACAGACGATT	Blocking

n0--K4-K6-	GGCCTTGAAGAGCCACCACCCTCAGAAACCAT	Blocking
n0--K6-K8-	CGATAGCATTGAGCCATTTGGGAACGTAGAAA	Blocking
n0--L3-L1-	TTTCGGAAGTGCCGTCGAGAGGGTGAGTTTCG	Blocking
n0--L13-L11-	GTACCGCAATTCTAAGAACGCGAGTATTATTT	Blocking
n0--L15-L13-	AATTGAGAATTCTGTCCAGACGACTAAACCAA	Blocking
n0--L17-L15-	ACCTTTTTATTTTAGTTAATTTCATAGGGCTT	Blocking
n0--L19-L17-	CGCGCAGATTACCTTTTTTAATGGGAGAGACT	Blocking
n0--L21-L19-	GCGGAACATCTGAATAATGGAAGGTACAAAAT	Blocking
n0--L23-L21-	AGCCAGCAATTGAGGAAGGTTATCATCATTTT	Blocking
n0--K24-L23-	ACCCTTCTGACCTGAAAGCGTAAGACGCTGAG	Blocking
n0--L5-L3-	CCACCCTCTATTCACAAACAAATACCTGCCTA	Blocking
n0--L7-L5-	TCACCGACGCACCGTAATCAGTAGCAGAACCG	Blocking
n0--L9-L7-	AAGGAAACATAAAGGTGGCAACATTATCACCG	Blocking
n0--L11-L9-	ATCCCAATGAGAATTAACTGAACAGTTACCAG	Blocking
n0--M8-M10-	AACGCAAAGATAGCCGAACAAACCCTGAAC	Blocking
n0--M10-M12-	AAAGTCACAAAATAAACAGCCAGCGTTTTA	Blocking
n0--M12-M14-	GCGAACCTCCAAGAACGGGTATGACAATAA	Blocking
n0--M14-M16-	ACAACATGCCAACGCTCAACAGTCTTCTGA	Blocking
n0--M16-M18-	CCTAAATCAAAATCATAGGTCTAAACAGTA	Blocking
n0--N1-M2-	AGGAACCCATGTACCGTAACACTTGATATAA	Blocking
n0--M18-M20-	CATAAATCTTTGAATACCAAGTGTTAGAAC	Blocking
n0--M20-M22-	CTACCATAGTTTGAGTAACATTTAAAATAT	Blocking
n0--M22-M24-	CTTTAGGGCCTGCAACAGTGCCAATACGTG	Blocking
n0--M2-M4-	GTATAGCAAACAGTTAATGCCCAATCCTCA	Blocking
n0--M4-M6-	TTAAAGCCAGAGCCGCCACCCTCGACAGAA	Blocking
n0--M6-M8-	TCAAGTTTCATTAAAGGTGAATATAAAAGA	Blocking
n0--N3-N1-	GCCCGTATCCGGAATAGGTGTATCAGCCCAAT	Blocking
n0--N13-N11-	CTTATCATTCCCGACTTGCGGGAGCCTAATTT	Blocking
n0--N15-N13-	AGTATAAAGTTCAGCTAATGCAGATGTCTTTC	Blocking
n0--N17-N15-	GAATTTATTTAATGGTTTGAAATATTCTTACC	Blocking
n0--N19-N17-	CCTGATTGCAATATATGTGAGTGATCAATAGT	Blocking
n0--N21-N19-	ATTTTAAAATCAAAATTATTTGCACGGATTCG	Blocking
n0--N23-N21-	TTAACACCAGCACTAACAACTAATCGTTATTA	Blocking
n0--M24-N23-	GCACAGACAATATTTTTGAATGGGGTCAGTA	Blocking
n0--N5-N3-	GCCTCCCTCAGAATGGAAAGCGCAGTAACAGT	Blocking
n0--N7-N5-	GAAATTATTGCCTTTAGCGTCAGACCGGAACC	Blocking
n0--N9-N7-	AAGTAAGCAGACACCACGGAATAATATTGACG	Blocking
n0--N11-N9-	GCCAGTTAGAGGGTAATTGAGCGCTTTAAGAA	Blocking
n0--O8-O10-	TGTCACAATCTTACCGAAGCCCTTTAATATCA	Blocking
n0--O10-O12-	GAGAGATAGAGCGTCTTTCCAGAGGTTTTGAA	Blocking
n0--O12-O14-	GCCTTAAACCAATCAATAATCGGCACGCGCCT	Blocking

n0--O14-O16-	GTTTATCAATATGCGTTATACAAACCGACCGT	Blocking
n0--O16-O18-	GTGATAAAAAGACGCTGAGAAGAGATAACCTT	Blocking
n0--P1-O2-	CCACCCTCATTTTCAGGGATAGCAACCGTACT	Blocking
n0--O18-O20-	GCTTCTGTTCGGGAGAAACAATAACGTAAAAC	Blocking
n0--O20-O22-	AGAAATAAAAATCCTTTGCCCGAAAGATTAGA	Blocking
n0--O22-O24-	GCCGTCAAAAAACAGAGGTGAGGCCTATTAGT	Blocking
n0--O2-O4-	CAGGAGGTGGGGTCAGTGCCTTGAGTCTCTGA	Blocking
n0--O4-O6-	ATTTACCGGGAACCAGAGCCACCACTGTAGCG	Blocking
n0--O6-O8-	CGTTTTCAAGGGAGGGAAGGTAAAGTTTATTT	Blocking
n0--P3-P1-	GTTTTAACTTAGTACCGCCACCCAGAGCCA	Blocking
n0--P13-P11-	TGTAGAAATCAAGATTAGTTGCTCTTACCA	Blocking
n0--P15-P13-	TTAGTATCACAATAGATAAGTCCACGAGCA	Blocking
n0--P17-P15-	CTTAGATTTAAGGCGTTAAATAAAGCCTGT	Blocking
n0--P19-P17-	CTTTTACAAAATCGTCGCTATTAGCGATAG	Blocking
n0--P21-P19-	CTCGTATTAGAAATTGCGTAGATACAGTAC	Blocking
n0--P23-P21-	CAGAAGATTAGATAATACATTTGTCGACAA	Blocking
n0--O24-P23-	CTTTAATGCGCGAACTGATAGCCCCACCAG	Blocking
n0--P5-P3-	AAATCACCTTCCAGTAAGCGTCAGTAATAA	Blocking
n0--P7-P5-	ACCGATTGTCGGCATTTTCGGTCATAATCA	Blocking
n0--P9-P7-	AATAGCTATCAATAGAAAATTCAACATTCA	Blocking
n0--P11-P9-	ACGCTAACACCCACAAGAATTGAAAATAGC	Blocking
n0--A2-A4-zJ	AGGCTCCAGAGGCTTTGAGGACACGGGTAATTATACATCTA	Docking
n0--A6-A8-zJ	CATCAAGTAAAACGAACTAACGAGTTGAGATTATACATCTA	Docking
n0--A10-A12-zJ	TTTACCCCAACATGTTTTAAATTTCCATATTTATACATCTA	Docking
n0--A14-A16-zJ	AACGCAAAATCGATGAACGGTACCGGTTGATTATACATCTA	Docking
n0--A18-A20-zJ	TGCATCTTTCCCAGTCACGACGGCCTGCAGTTATACATCTA	Docking
n0--A22-A24-zJ	TTTTCACTCAAAGGGCGAAAAACCATCACCTTATACATCTA	Docking
n0--C2-C4-zJ	TTTATCAGGACAGCATCGGAACGACACCAACCTTATACATCTA	Docking
n0--C6-C8-zJ	TTCATTACGTCAGGACGTTGGGAAATGCAGATTTATACATCTA	Docking
n0--C10-C12-zJ	CGGATTGCAGAGCTTAATTGCTGAAACGAGTATTATACATCTA	Docking
n0--C14-C16-zJ	TATATTTTGTCATTGCCTGAGAGTGGAAGATTTTATACATCTA	Docking
n0--C18-C20-zJ	TATCGGCCGCAAGGCGATTAAGTTTACCGAGCTTATACATCTA	Docking
n0--C22-C24-zJ	GCCCTTCAGAGTCCACTATTAAAGGGTGCCGTTTATACATCTA	Docking
n0--E2-E4-zJ	AAACAGCTTTTTGCGGGATCGTCAACACTAAATTATACATCTA	Docking
n0--E6-E8-zJ	TCATTCAGATGCGATTTTAAGAACAGGCATAGTTATACATCTA	Docking
n0--E10-E12-zJ	CGAAAGACTTTGATAAGAGGTCATATTTCGCATTATACATCTA	Docking
n0--E14-E16-zJ	TAGGTAAACTATTTTTGAGAGATCAAACGTTATTATACATCTA	Docking
n0--E18-E20-zJ	GCTTTCCGATTACGCCAGCTGGCGGCTGTTTCTTATACATCTA	Docking
n0--E22-E24-zJ	AGCAAGCGTAGGGTTGAGTGTTGTAGGGAGCCTTATACATCTA	Docking
n0--G2-G4-zJ	TGACAACTCGCTGAGGCTTGCATTATACCATTATACATCTA	Docking

n0--G6-G8-zJ	AACACCAAATTTCAACTTTAATCGTTTACCTTATACATCTA	Docking
n0--G10-G12-zJ	GCTTCAATCAGGATTAGAGAGTTATTTTCATTATACATCTA	Docking
n0--G14-G16-zJ	GAGACAGCTAGCTGATAAATTAATTTTTGTTTATACATCTA	Docking
n0--G18-G20-zJ	AGGCAAAGGGAAGGGCGATCGGCAATTCCATTATACATCTA	Docking
n0--G22-G24-zJ	GCGAAAAATCCCTTATAAATCAAGCCGGCGTTATACATCTA	Docking
n0--H5-I4-zJ	TCATCGCCAACAAAGTACAACGGACGCCAGCATTATACATCTA	Docking
n0--H9-I8-zJ	CTTTTGCAGATAAAAACCAAAATAAAGACTCCTTATACATCTA	Docking
n0--H13-I12-zJ	TTCTACTACGCGAGCTGAAAAGGTTACCGCGCTTATACATCTA	Docking
n0--H17-I16-zJ	GCCATCAAGCTCATTTTTTAACCACAAATCCATTATACATCTA	Docking
n0--H21-I20-zJ	AAGCCTGGTACGAGCCGGAAGCATAGATGATGTTATACATCTA	Docking
n0--G24-I24-zJ	AACGTGGCGAGAAAGGAAGGGAAACCAGTAATTATACATCTA	Docking
n0--K2-K4-zJ	GCGGATAACCTATTATTCTGAAACAGACGATTTTATACATCTA	Docking
n0--K6-K8-zJ	CGATAGCATTGAGCCATTTGGGAACGTAGAAATTATACATCTA	Docking
n0--K10-K12-zJ	TTAGACGGCCAAATAAGAAACGATAGAAGGCTTTATACATCTA	Docking
n0--K14-K16-zJ	GTAAAGTAATCGCCATATTTAACAAAACTTTTTTATACATCTA	Docking
n0--K18-K20-zJ	CATTTGAAGGCGAATTATTCATTTTTGTTTGGTTATACATCTA	Docking
n0--K22-K24-zJ	TGAAAGGAGCAAATGAAAAATCTAGAGATAGATTATACATCTA	Docking
n0--M2-M4-zJ	GTATAGCAAACAGTTAATGCCCAATCCTCATTATACATCTA	Docking
n0--M6-M8-zJ	TCAAGTTTCATTAAAGGTGAATATAAAAGATTATACATCTA	Docking
n0--M10-M12-zJ	AAAGTCACAAAATAAACAGCCAGCGTTTTATTATACATCTA	Docking
n0--M14-M16-zJ	ACAACATGCCAACGCTCAACAGTCTTCTGATTATACATCTA	Docking
n0--M18-M20-zJ	CATAAATCTTTGAATACCAAGTGTTAGAACTTATACATCTA	Docking
n0--M22-M24-zJ	CTTTAGGGCCTGCAACAGTGCCAATACGTGTTATACATCTA	Docking
n0--O2-O4-zJ	CAGGAGGTGGGGTCAGTGCCTTGAGTCTCTGATTATACATCTA	Docking
n0--O6-O8-zJ	CGTTTTCAAGGGAGGGAAGGTAAAGTTTATTTTTATACATCTA	Docking
n0--O10-O12-zJ	GAGAGATAGAGCGTCTTTCCAGAGGTTTTGAATTATACATCTA	Docking
n0--O14-O16-zJ	GTTTATCAATATGCGTTATACAAACCGACCGTTTATACATCTA	Docking
n0--O18-O20-zJ	GCTTCTGTTCGGGAGAAACAATAACGTAAAACTTATACATCTA	Docking
n0--O22-O24-zJ	GCCGTCAAAAAACAGAGGTGAGGCCTATTAGTTTATACATCTA	Docking

Table S1. dNAM Staple Strands

The staple strand name indicates the structure version (n0: a NAM-modified version of Jungmann's rectangle), the 5' end modification (ptt: passivation, polythymine for registration strands), the 5' end position, the 3' end position (see staple strand position map below), and the 3' end modification (z: 3' oriented dock; J: 3' Mid dock, for Docking strands).

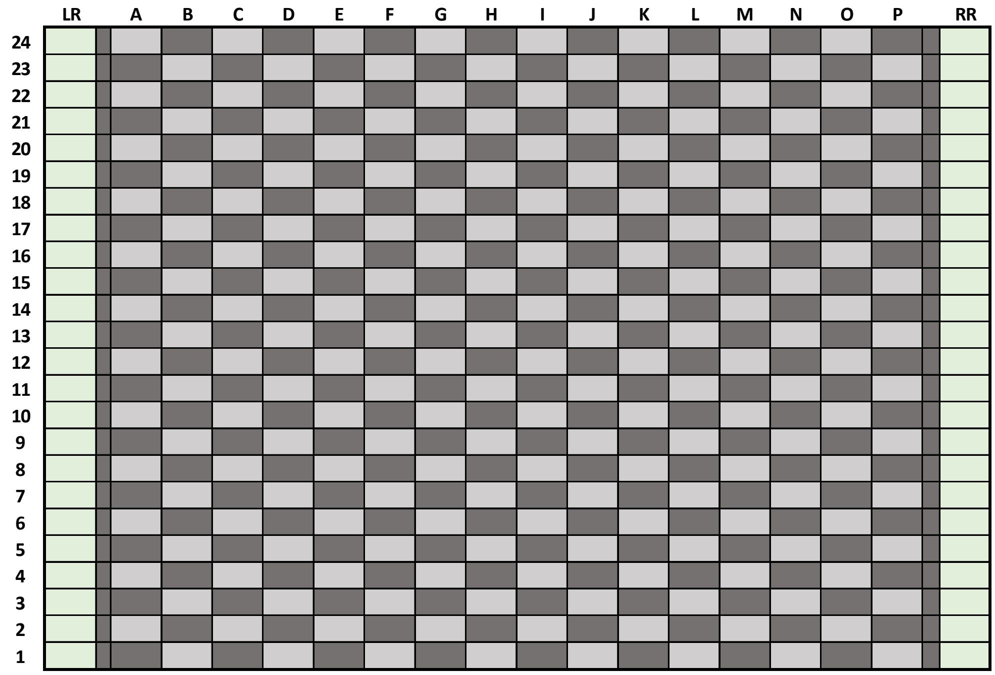

Table S2. dNAM Staple Strand Position Map

The domains (A-P, LR, and RR) and helices (1-24) used to locate the staple strands are depicted.

Name	Sequence	Modification
A01	CGGGGTTTCCTCAAGAGAAGGATTTTGAATTA	
A02	AGCGTCATGTCTCTGAATTTACCGACTACCTT	
A03	TTCATAATCCCCTTATTAGCGTTTTTCTTACC	
A04	ATGGTTTATGTCACAATCAATAGATATTAAAC	
A05	TTTGATGATTAAGAGGCTGAGACTTGCTCAGTACCAGGCG	
A06	CCGGAACCCAGAATGGAAAGCGCAACATGGCT	
A07	AAAGACAACATTTTCGGTCATAGCCAAAATCA	
A08	GACGGGAGAATTAACTCGGAATAAGTTTATTTCCAGCGCC	
A09	GATAAGTGCCGTCGAGCTGAAACATGAAAGTATACAGGAG	
A10	TGTACTGGAAATCCTCATTAAAGCAGAGCCAC	
A11	CACCGGAAAGCGCGTTTTCATCGGAAGGGCGA	
A12-TT-Mid	CATTCAACAAACGCAAAGACACCAGAACACCCTGAACAAATTATACATCT	Docking site
A13	TTTAACGGTTCGGAACCTATTATTAGGGTTGATATAAGTA	
A14	CTCAGAGCATATTCACAAACAAATTAATAAGT	
A15	GGAGGGAATTTAGCGTCAGACTGTCCGCCTCC	
A16	GTCAGAGGGTAATTGATGGCAACATATAAAAGCGATTGAG	
A17	TAGCCCGGAATAGGTGAATGCCCCCTGCCTATGGTCAGTG	
A18	CCTTGAGTCAGACGATTGGCCTTGCGCCACCC	
A19	TCAGAACCCAGAATCAAGTTTGCCGGTAAATA	
A20	TTGACGGAAATACATACATAAAGGGCGCTAATATCAGAGA	
A21	CAGAGCCAGGAGGTTGAGGCAGGTAACAGTGCCCG	
A22	ATTAAAGGCCGTAATCAGTAGCGAGCCACCCT	
A23	GATAACCCACAAGAATGTTAGCAAACGTAGAAAATTATTC	
A24	GCCGCCAGCATTGACACCACCCTC	
A25	AGAGCCGCACCATCGATAGCAGCATGAATTAT	
A26	CACCGTCACCTTATTACGCAGTATTGAGTTAAGCCCAATA	
A27	AGCCATTTAAACGTCACCAATGAACACCAGAACCA	
A28	ATAAGAGCAAGAAACATGGCATGATTAAGACTCCGACTTG	
A29	CCATTAGCAAGGCCGGGGGAATTA	
A30-TT-Mid	GAGCCAGCGAATACCCAAAAGAACATGAAATAGCAATAGCTTATACATCT	Docking site
A31	TATCTTACCGAAGCCCAAACGCAATAATAACGAAAATCACCAG	
A32	CAGAAGGAAACCGAGGTTTTTAAGAAAAGTAAGCAGATAGCCG	
A33	CCTTTTTTCATTTAACAATTTCATAGGATTAG	
A34	TTTAACCTATCATAGGTCTGAGAGTTCCAGTA	
A35	AGTATAAAATATGCGTTATACAAAGCCATCTT	
A36	CAAGTACCTCATTCCAAGAACGGGAAATTCAT	
A37	AGAGAATAACATAAAAACAGGGAAGCGCATTA	
A38	AAAACAAAATTAATTAAATGGAAACAGTACATTAGTGAAT	
A39	TTATCAAACCGGCTTAGGTTGGGTAAGCCTGT	
A40	TTAGTATCGCCAACGCTCAACAGTCGGCTGTC	
A41	TTTCCTTAGCACTCATCGAGAACAATAGCAGCCTTTACAG	
A42	AGAGTCAAAAATCAATATATGTGATGAAACAAACATCAAG	
A43	ACTAGAAATATATAACTATATGTACGCTGAGA	

A44	TCAATAATAGGGCTTAATTGAGAATCATAATT	
A45	AACGTCAAAAATGAAAAGCAAGCCGTTTTTATGAAACCAA	
A46	GAGCAAAAGAAGATGAGTGAATAACCTTGCTTATAGCTTA	
A47	GATTAAGAAATGCTGATGCAAATCAGAATAAA	
A48	CACCGGAATCGCCATATTTAACAAAATTTACG	
A49-TT-Mid	AGCATGTATTTCATCGTAGGAATCAAACGATTTTTTGTTTTTATACATCT	Docking site
A50	ACATAGCGCTGTAAATCGTCGCTATTCATTTCAATTACCT	
A51	GTTAAATACAATCGCAAGACAAAGCCTTGAAA	
A52	CCCATCCTCGCCAACATGTAATTTAATAAGGC	
A53	TCCCAATCCAAATAAGATTACCGCGCCCAATAAATAATAT	
A54	TCCCTTAGAATAACGCGAGAAAACTTTTACCGACC	
A55	GTGTGATAAGGCAGAGGCATTTTCAGTCCTGA	
A56	ACAAGAAAGCAAGCAAATCAGATAACAGCCATATTATTTA	
A57	GTTTGAAATTCAAATATATTTTAG	
A58	AATAGATAGAGCCAGTAATAAGAGATTTAATG	
A59	GCCAGTTACAAAATAATAGAAGGCTTATCCGGTTATCAAC	
A60	TTCTGACCTAAAATATAAAGTACCGACTGCAGAAC	
A61	GCGCCTGTTATTCTAAGAACGCGATTCCAGAGCCTAATTT	
A62	TCAGCTAAAAAAGGTAAAGTAATT	
A63	ACGCTAACGAGCGTCTGGCGTTTTAGCGAACCCAACATGT	
A64-TT-Mid	ACGACAATAAATCCCGACTTGCGGGAGATCCTGAATCTTACCATTATACATCT	Docking site
A65	TGCTATTTTGCACCCAGCTACAATTTTGTTTTGAAGCCTTAAA	
B01	TCATATGTGTAATCGTAAAACTAGTCATTTTC	
B02	GTGAGAAAATGTGTAGGTAAAGATACAACTTT	
B03	GGCATCAAATTTGGGGCGCGAGCTAGTTAAAG	
B04	TTCGAGCTAAGACTTCAAATATCGGGAACGAG	
B05	ACAGTCAAAGAGAATCGATGAACGACCCCGGTTGATAATC	
B06	ATAGTAGTATGCAATGCCTGAGTAGGCCGGAG	
B07	AACCAGACGTTTAGCTATATTTTCTTCTACTA	
B08	GAATACCACATTCAACTTAAGAGGAAGCCCGATCAAAGCG	
B09	AGAAAAGCCCCAAAAAGAGTCTGGAGCAAACAATCACCAT	
B10	CAATATGACCCTCATATATTTTAAAGCATTAA	
B11	CATCCAATAAATGGTCAATAACCTCGGAAGCA	
B12-TT-Mid	AACTCCAAGATTGCATCAAAAAGATAATGCAGATACATAATTATACATCT	Docking site
B13	CGTTCTAGTCAGGTCATTGCCTGACAGGAAGATTGTATAA	
B14	CAGGCAAGATAAAAATTTTTAGAATATTCAAC	
B15	GATTAGAGATTAGATACATTTCGCAAATCATA	
B16	CGCCAAAAGGAATTACAGTCAGAAGCAAAGCGCAGGTCAG	
B17	GCAAATATTTAAATTGAGATCTACAAAGGCTACTGATAAA	
B18	TTAATGCCTTATTTCAACGCAAGGGCAAAGAA	
B19	TTAGCAAATAGATTTAGTTTGACCAGTACCTT	
B20	TAATTGCTTTACCCTGACTATTATGAGGCATAGTAAGAGC	
B21	ATAAAGCCTTTGCGGGAGAAGCCTGGAGAGGGTAG	
B22	TAAGAGGTCAATTCTGCGAACGAGATTAAGCA	

B23	AACACTATCATAACCCATCAAAAATCAGGTCTCCTTTTGA	
B24	ATGACCCTGTAATACTTCAGAGCA	
B25	TAAAGCTATATAACAGTTGATTCCCATTTTTG	
B26	CGGATGGCACGAGAATGACCATAATCGTTTACCAGACGAC	
B27	TAATTGCTTGGAAGTTTCATTCCAAATCGGTTGTA	
B28	GATAAAAACCAAAATATTAAACAGTTCAGAAATTAGAGCT	
B29	ACTAAAGTACGGTGTCGAATATAA	
B30-TT-Mid	TGCTGTAGATCCCCCTCAAATGCTGCGAGAGGCTTTTGCATTATACATCT	Docking site
B31	AAAGAAGTTTTGCCAGCATAAATATTCATTGACTCAACATGTT	
B32	AATACTGCGGAATCGTAGGGGGTAATAGTAAAATGTTTAGACT	
B33	AGGGATAGCTCAGAGCCACCACCCCATGTCAA	
B34	CAACAGTTTATGGGATTTTGCTAATCAAAAGG	
B35	GCCGCTTTGCTGAGGCTTGCAGGGGAAAAGGT	
B36	GCGCAGACTCCATGTTACTTAGCCCGTTTTAA	
B37	ACAGGTAGAAAGATTCATCAGTTGAGATTTAG	
B38	CCTCAGAACCGCCACCCAAGCCCAATAGGAACGTAAATGA	
B39	ATTTTCTGTCAGCGGAGTGAGAATACCGATAT	
B40	ATTCGGTCTGCGGGATCGTCACCCGAAATCCG	
B41	CGACCTGCGGTCAATCATAAGGGAACGGAACAACATTATT	
B42	AGACGTTACCATGTACCGTAACACCCCTCAGAACCGCCAC	
B43	CACGCATAAGAAAGGAACAACTAAGTCTTTCC	
B44	ATTGTGTCTCAGCAGCGAAAGACACCATCGCC	
B45	TTAATAAAACGAACTAACCGAACTGACCAACTCCTGATAA	
B46	AGGTTTAGTACCGCCATGAGTTTCGTCACCAGGATCTAAA	
B47	GTTTTGTCAGGAATTGCGAATAATCCGACAAT	
B48	GACAACAAGCATCGGAACGAGGGTGAGATTTG	
B49-TT-Mid	TATCATCGTTGAAAGAGGACAGATGGAAGAAAAATCTACGTTATACATCT	Docking site
B50	AGCGTAACTACAAACTACAACGCCTATCACCGTACTCAGG	
B51	TAGTTGCGAATTTTTTCACGTTGATCATAGTT	
B52	GTACAACGAGCAACGGCTACAGAGGATACCGA	
B53	ACCAGTCAGGACGTTGGAACGGTGTACAGACCGAAACAAA	
B54	ACAGACAGCCCAAATCTCCAAAAAAAAATTTCTTA	
B55	AACAGCTTGCTTTGAGGACTAAAGCGATTATA	
B56	CCAAGCGCAGGCGCATAGGCTGGCAGAACTGGCTCATTAT	
B57	CGAGGTGAGGCTCCAAAAGGAGCC	
B58	ACCCCCAGACTTTTTCATGAGGAACTTGCTTT	
B59	ACCTTATGCGATTTTATGACCTTCATCAAGAGCATCTTTG	
B60	CGGTTTATCAGGTTTCCATTAAACGGGAATACACT	
B61	AAAACACTTAATCTTGACAAGAACTTAATCATTGTGAATT	
B62	GGCAAAAGTAAAATACGTAATGCC	
B63	TGGTTTAATTTCAACTCGGATATTCATTACCCACGAAAGA	
B64-TT-Mid	ACCAACCTAAAAAATCAACGTAACAAATAAATTGGGCTTGAGATTATACATCT	Docking site
B65	CCTGACGAGAAACACCAGAACGAGTAGGCTGCTCATTCAGTGA	
C01	TCGGGAGATATACAGTAACAGTACAAATAATT	

C02	CCTGATTAAAGGAGCGGAATTATCTCGGCCTC	
C03	GCAAATCACCTCAATCAATATCTGCAGGTCGA	
C04	CGACCAGTACATTGGCAGATTCACCTGATTGC	
C05	TGGCAATTTTTAACGTCAGATGAAAACAATAACGGATTCG	
C06	AAGGAATTACAAAGAAACCACCAGTCAGATGA	
C07	GGACATTCACCTCAAATATCAAACACAGTTGA	
C08	TTGACGAGCACGTATACTGAAATGGATTATTTAATAAAAG	
C09	CCTGATTGCTTTGAATTGCGTAGATTTTCAGGCATCAATA	
C10	TAATCCTGATTATCATTTTGCGGAGAGGAAGG	
C11	TTATCTAAAGCATCACCTTGCTGATGGCCAAC	
C12-TT-Mid	AGAGATAGTTTGACGCTCAATCGTACGTGCTTTCCTCGTTTTATACATCT	Docking site
C13	GATTATACACAGAAATAAAGAAATACCAAGTTACAAAATC	
C14	TAGGAGCATAAAAGTTTGAGTAACATTGTTTG	
C15	TGACCTGACAAATGAAAAATCTAAAATATCTT	
C16	AGAATCAGAGCGGGAGATGGAAATACCTACATAACCCTTC	
C17	GCGCAGAGGCGAATTAATTATTTGCACGTAAATTCTGAAT	
C18	AATGGAAGCGAACGTTATTAATTTCTAACAAC	
C19	TAATAGATCGCTGAGAGCCAGCAGAAGCGTAA	
C20	GAATACGTAACAGGAAAAACGCTCCTAAACAGGAGGCCGA	
C21	TCAATAGATATTAAATCCTTTGCCGGTTAGAACCT	
C22	CAATATTTGCCTGCAACAGTGCCATAGAGCCG	
C23	TTAAAGGGATTTTAGATACCGCCAGCCATTGCGGCACAGA	
C24	ACAATTCGACAACTCGTAATACAT	
C25	TTGAGGATGGTCAGTATTAACACCTTGAATGG	
C26	CTATTAGTATATCCAGAACAATATCAGGAACGGTACGCCA	
C27	CGCGAACTAAAACAGAGGTGAGGCTTAGAAGTATT	
C28	GAATCCTGAGAAGTGTATCGGCCTTGCTGGTACTTTAATG	
C29	ACCACCAGCAGAAGATGATAGCCC	
C30-TT-Mid	TAAAACATTAGAAGAACTCAAACTTTTTATAATCAGTGAGTTATACATCT	Docking site
C31	GCCACCGAGTAAAAGAACATCACTTGCCTGAGCGCCATTAAAA	
C32	TCTTTGATTAGTAATAGTCTGTCCATCACGCAAATTAACCGTT	
C33	CGCGTCTGATAGGAACGCCATCAACTTTTACA	
C34	AGGAAGATGGGGACGACGACAGTAATCATATT	
C35	CTCTAGAGCAAGCTTGCATGCCTGGTCAGTTG	
C36	CCTTCACCGTGAGACGGGCAACAGCAGTCACA	
C37	CGAGAAAGGAAGGGAAGCGTACTATGGTTGCT	
C38	GCTCATTTTTTAACCAGCCTTCCTGTAGCCAGGCATCTGC	
C39	CAGTTTGACGCACTCCAGCCAGCTAAACGACG	
C40	GCCAGTGCGATCCCCGGGTACCGAGTTTTTCT	
C41	TTTCACCAGCCTGGCCCTGAGAGAAAGCCGGCGAACGTGG	
C42	GTAACCGTCTTTCATCAACATTAAAATTTTTGTTAAATCA	
C43	ACGTTGTATTCCGGCACCGCTTCTGGCGCATC	
C44	CCAGGGTGGCTCGAATTCGTAATCCAGTCACG	
C45	TAGAGCTTGACGGGGAGTTGCAGCAAGCGGTCATTGGGCG	

C46	GTTAAAATTCGCATTAATGTGAGCGAGTAACACACGTTGG	
C47	TGTAGATGGGTGCCGGAAACCAGGAACGCCAG	
C48	GGTTTTCCATGGTCATAGCTGTTTGAGAGGCG	
C49-TT-Mid	GTTTGCGTCACGCTGGTTTGCCCCAAGGGAGCCCCCGATTTTATACATCT	Docking site
C50	GGATAGGTACCCGTCGGATTCTCCTAAACGTTAATATTTT	
C51	AGTTGGGTCAAAGCGCCATTCGCCCCGTAATG	
C52	CGCGCGGGCCTGTGTGAAATTGTTGGCGATTA	
C53	CTAAATCGGAACCCTAAGCAGGCGAAAATCCTTCGGCCAA	
C54	CGGCGGATTGAATTCAGGCTGCGCAACGGGGGATG	
C55	TGCTGCAAATCCGCTCACAATTCCCAGCTGCA	
C56	TTAATGAAGTTTGATGGTGGTTCCGAGGTGCCGTAAAGCA	
C57	TGGCGAAATGTTGGGAAGGGCGAT	
C58	TGTCGTGCACACAACATACGAGCCACGCCAGC	
C59	CAAGTTTTTTGGGGTCGAAATCGGCAAAATCCGGGAAACC	
C60	TCTTCGCTATTGGAAGCATAAAGTGTATGCCCGCT	
C61	TTCCAGTCCTTATAAATCAAAAGAGAACCATCACCCAAAT	
C62	GCGCTCACAAGCCTGGGGTGCCTA	
C63	CGATGGCCCACTACGTATAGCCCGAGATAGGGATTGCGTT	
C64-TT-Mid	AACTCACATTATTGAGTGTTGTTCCAGAAACCGTCTATCAGGGTTATACATCT	Docking site
C65	ACGTGGACTCCAACGTCAAAGGGCGAATTTGGAACAAGAGTCC	
Link-A1C	TTAATTAATTTTTTACCATATCAAA	
Link-A2C	TTAATTTCATCTTAGACTTTACAA	
Link-A3C	CTGTCCAGACGTATACCGAACGA	
Link-A4C	TCAAGATTAGTGTAGCAATACT	
Link-B1A	TGTAGCATTCCTTTTATAAACAGTT	
Link-B2A	TTTAATTGTATTTCCACCAGAGCC	
Link-B3A	ACTACGAAGGCTTAGCACCATTA	
Link-B4A	ATAAGGCTTGCAACAAAGTTAC	
Link-C1B	GTGGGAACAAATTTCTATTTTTGAG	
Link-C2B	CGGTGCGGGCCTTCCAAAAACATT	
Link-C3B	ATGAGTGAGCTTTTAAATATGCA	
Link-C4B	ACTATTAAAGAGGATAGCGTCC	
Loop	GCGCTTAATGCGCCGCTACAGGGC	

Table S3. Triangle Staple Strands

The oligonucleotide sequences used for the sharp triangle origami design (used in the study as a fiducial marker) are indicated.

Encoding/Decoding Algorithms

See attached diagrams and flowcharts for graphical representation of the main steps of the algorithms. **Table S4** lists the different designs generated by the encoding algorithm for the message 'Data is in our DNA!\n'.

Index	Binary Index	Droplet	Matrix Design	Index	Binary Index	Droplet	Matrix Design	Index	Binary Index	Droplet	Matrix Design
**0**	0000	00110110<br>01010101	00110110<br>11110111<br>00111010<br>00110111<br>11011110<br>00101010	**5**	0101	01011111<br>01001011	01011111<br>11010101<br>10111000<br>00101001<br>11000010<br>10110100	**10**	1010	01101110<br>00000101	01101110<br>11010101<br>00000110<br>11011110<br>11111100<br>01101000
**1**	0001	00100110<br>01100000	00100110<br>10101001<br>10110100<br>00011101<br>11111100<br>00000001	**6**	0110	00010000<br>01101001	00010000<br>11101011<br>00011010<br>11100011<br>10000110<br>10100101	**11**	1011	00010001<br>00001010	00010001<br>11100111<br>10011010<br>10000000<br>10101110<br>01010100
**2**	0010	01011111<br>00111010	01011111<br>11011001<br>00011100<br>10111100<br>11100100<br>00010111	**7**	0111	01010100<br>00001000	01010100<br>10100001<br>11100000<br>11011000<br>11011000<br>10000100	**12**	1100	01001110<br>01000110	01001110<br>11001101<br>01000100<br>00000001<br>11000100<br>11011000
**3**	0011	00100001<br>01010110	00100001<br>10000101<br>10011110<br>10010001<br>11100010<br>00011010	**8**	1000	00100000<br>01101001	00100000<br>10111111<br>01000100<br>01010011<br>11100100<br>01100101	**13**	1101	01010010<br>01110110	01010010<br>10011111<br>11001100<br>01010011<br>11001110<br>11011011
**4**	0100	00011010<br>00010010	00011010<br>11111011<br>00111110<br>00010000<br>11011000<br>10010010	**9**	1001	00100000<br>01101111	00100000<br>11011011<br>10101100<br>00010011<br>11010000<br>01111101	**14**	1110	00001010<br>01111101	00001010<br>11010101<br>01101100<br>10001011<br>10010100<br>11101111

Table S4. Origami Designs

The binary data droplets and data strings associated with each origami index are shown.

Atomic Force Microscopy

AFM analysis was conducted on freshly cut mica substrates or glass coverslips (prepared as described above). 4 µL of a dNAM origami sample was deposited onto the substrate for 5 min and then 100 µL of deposition buffer added to form a droplet on top of the sample. AFM imaging was performed with a Dimension-FastScan system from Bruker set to amplitude modulation mode. Imaging was carried out in liquid with a set-point ratio between the free amplitude and imaging amplitude of ~0.7. The FastScan D cantilever was supplied by Bruker, with a nominal spring constant of 0.25 N/m. Sub-nanometer amplitude was used to image DNA docking strand positions on every origami structure following the method of[1]. Tilt correction (line or plane flattening) was performed using WSxM software package version 5.0 Develop 9.2[2] (Nanotec Electronica, Madrid, Spain) and a low-pass filter applied to remove noise. Further filtering, using inverse FFT band rejection, was added to visually highlight the docking strands.

Supplementary Results

DNA-PAINT Resolution

To evaluate the resolution of the DNA-PAINT experiments, FWHM values were derived by taking transect measurements centered on binding sites in rendered images (with 1-pixel blur applied) of either individual or 'averaged' dNAM origami (**Fig. S1**). In both cases at least ten binding sites were examined for each structure using with horizontally or vertically aligned positioned transects (**Fig. S1 a,b**). FWHM values of 6.6 nm ± 1.6 SD (single origami images, n = 124) and 7.2 nm ± 0.3 SD (averaged origami images, n = 47) were calculated from Gaussian fits to plots of the transect data (**Fig. S1 c,d**).

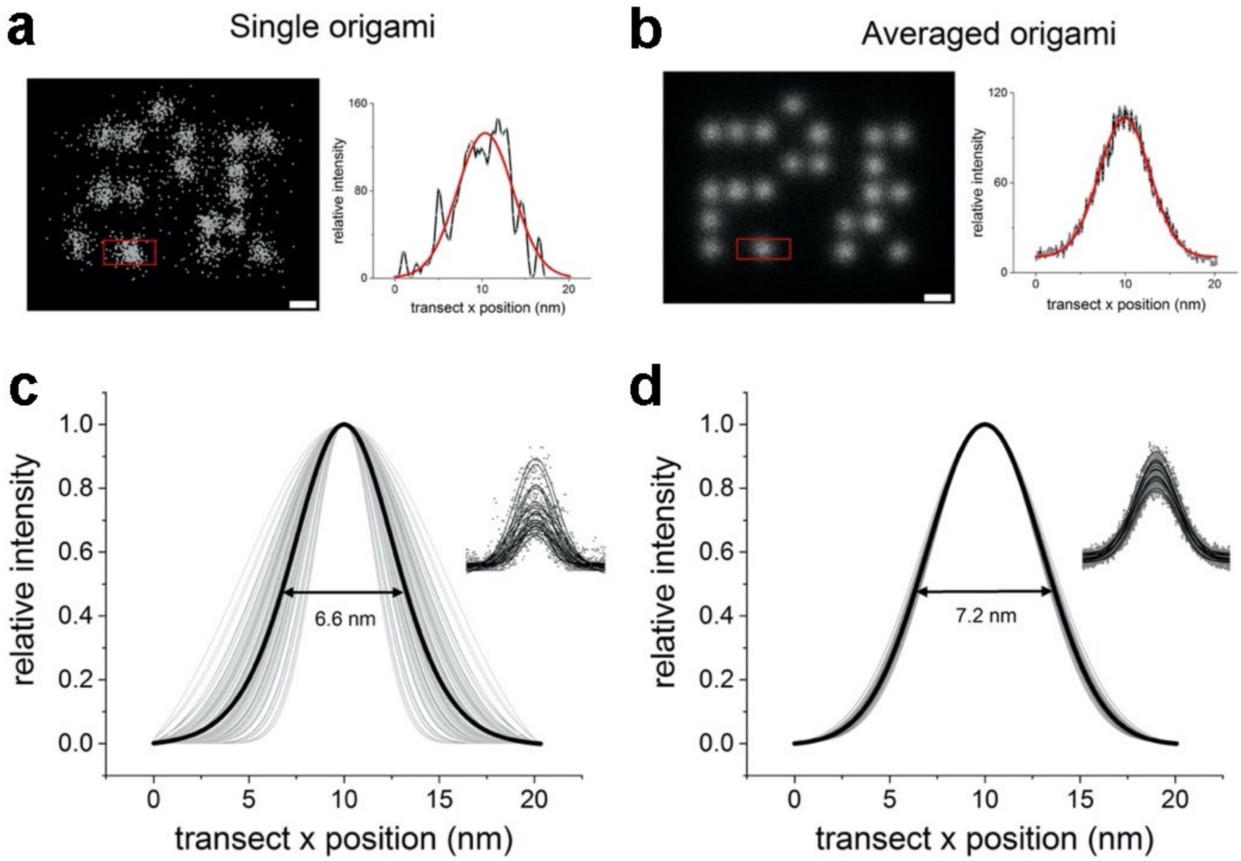

Figure S1. DNA-PAINT imaging of dNAM origami demonstrates low nm resolution for 'averaged' and individual structures

FWHM values were derived by taking transect measurements centered on binding sites in rendered images of either individual (**a**) or 'averaged' dNAM origami (**b**). Transects were placed horizontally (as shown in red) on vertically for measurements. A plot from a single binding site is shown with a Gaussian fit to the data plotted in red. Gaussian fits for binding sites from each experiment are plotted in grey for both single (**c**) and 'averaged' (**d**) structures (after centering and normalization). The mean of the grey lines is shown in black. The inset plots are the representative results from a single experimental recording. The mean FWHM value for individual fits to each experiment was calculated and reported in the main text. Origami-6 was used in all cases, as it was the most consistently recovered structure. (**a-b**) Scale bars, 10 nm. Single examples are shown. (**c-d**) n = 124 (single) and 47 (averaged) sites over 3 independent DNA-PAINT recordings.

Proximity Error Analysis

Analysis of our error locations (**Fig. S2**) showed slightly higher false negative error rates around the edges of dNAM origami, but there was no pattern of error locations in the origami that would explain the variance in error rates between different origami designs. There is a correlation between a higher number of 1-bits and a higher number of false negatives, as would be expected, but this does not explain most of the observed variance between origami. The phenomenon of higher errors near the edges of the origami has been observed previously[3] and was interpreted as reflecting a difference in staple strand incorporation efficiencies. To investigate this and other sources of potential sources of error in our array designs, we performed atomic force microscopy (AFM) imaging on individual origami deposited on mica (**Fig. S3**). From the averaged SRM images in **Fig. 2**, it can be seen that every data strand was recorded at least once for all expected positions in all arrays. This suggests that there were no systematic failures in strand incorporation or data strand binding domains. This is further substantiated by the AFM images, in which origami were typically both well formed (lacking holes and having the expected dimensions) and appeared to have incorporated the majority of their data strands. Although it was possible to resolve the majority of data strands positions (**Fig. S3**), a strict analysis on missing data strands using AFM would not be completely reliable since tip-sample interactions could easily promote strand compression and displacement. However, our previous correlative defect analysis of DNA origami, combining AFM and DNA-PAINT, indicated that strand incorporation plays a role in origami site yields and defects are likely due to the unavailability of incorporated staple strands. Further, DNA-PAINT itself may locally increase the susceptibility of DNA origami to damage during imaging[4]. This is in keeping with our results and suggests that further optimization of the DNA-PAINT imaging protocol will help reduce the false negative error rate.

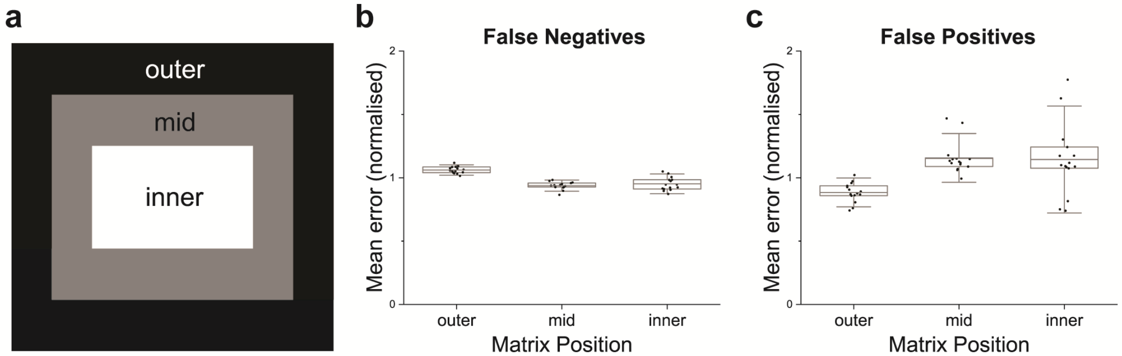

Figure S2. The outer edges and inner regions of dNAM origami are differentially error prone

The array positions of DNA origami (only considering structures with 15 or less errors, as identified by template matching) were classified as either ‘outer’, ‘mid’ or ‘inner’ depending on their position in the array (**a**). The mean error for each classification was calculated and normalized by dividing by the overall mean error for that zone. Plots of the mean normalized false negative (**b**) and false positive values (**c**) for each zone are shown. Individual data points plot the mean errors for each origami design. The box plots depict mean values and the 25-75% range, with whiskers plotting the SD. n = 15 origami designs over 3 DNA-PAINT recordings.

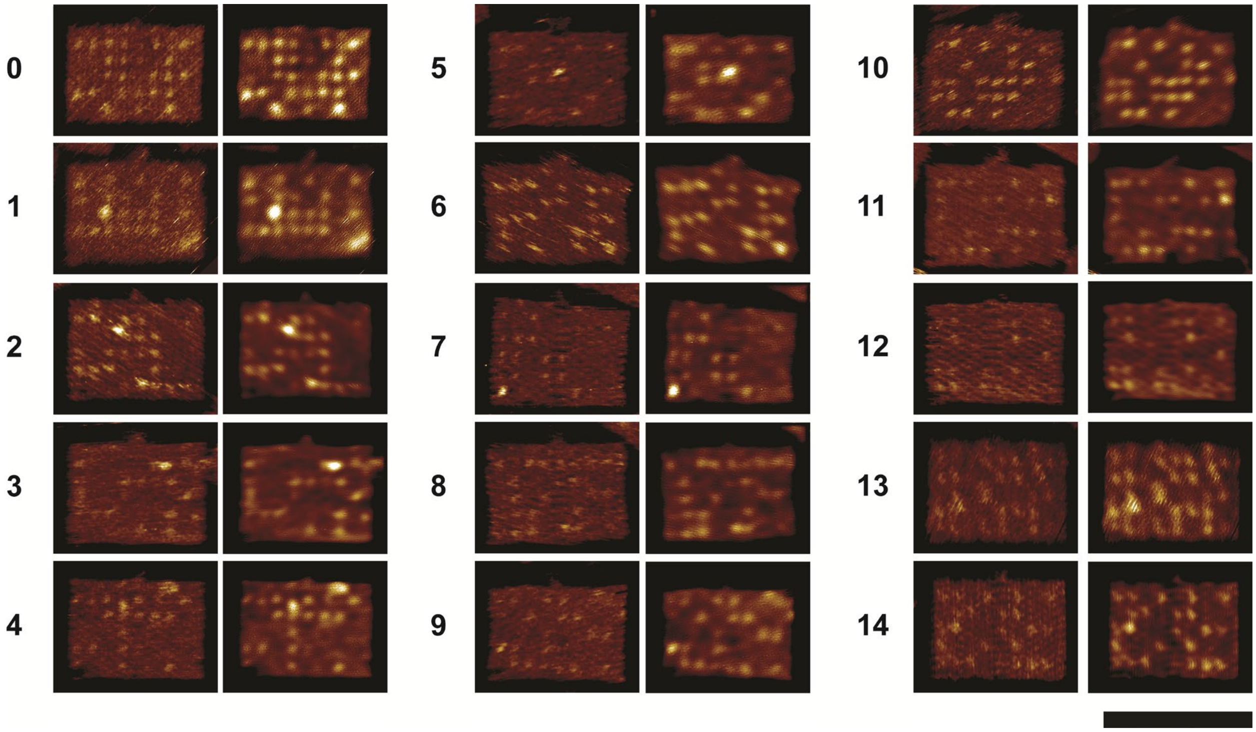

Figure S3. AFM images of dNAM origami

Representative AFM images of all 15 dNAM “Data is in our DNA!/n” origami, where most of dockings sites are visible. (An inverse FFT analysis with a band rejected filter has been applied to highlight the docking positions in right-hand panels). Every image is 90 x110 nm^2 and the color scale ranges over 250 pm. Black scale bar is 100 nm.

Supplementary Diagrams and Charts

Figure S4. The Encoding Algorithm

The multi-page figure below illustrates the twelve steps involved in encoding a text message using dNAM. The encoding process depicts the proof-of-principle experiment described in the main text, showing the design process for one of the 15 origami, as an example.

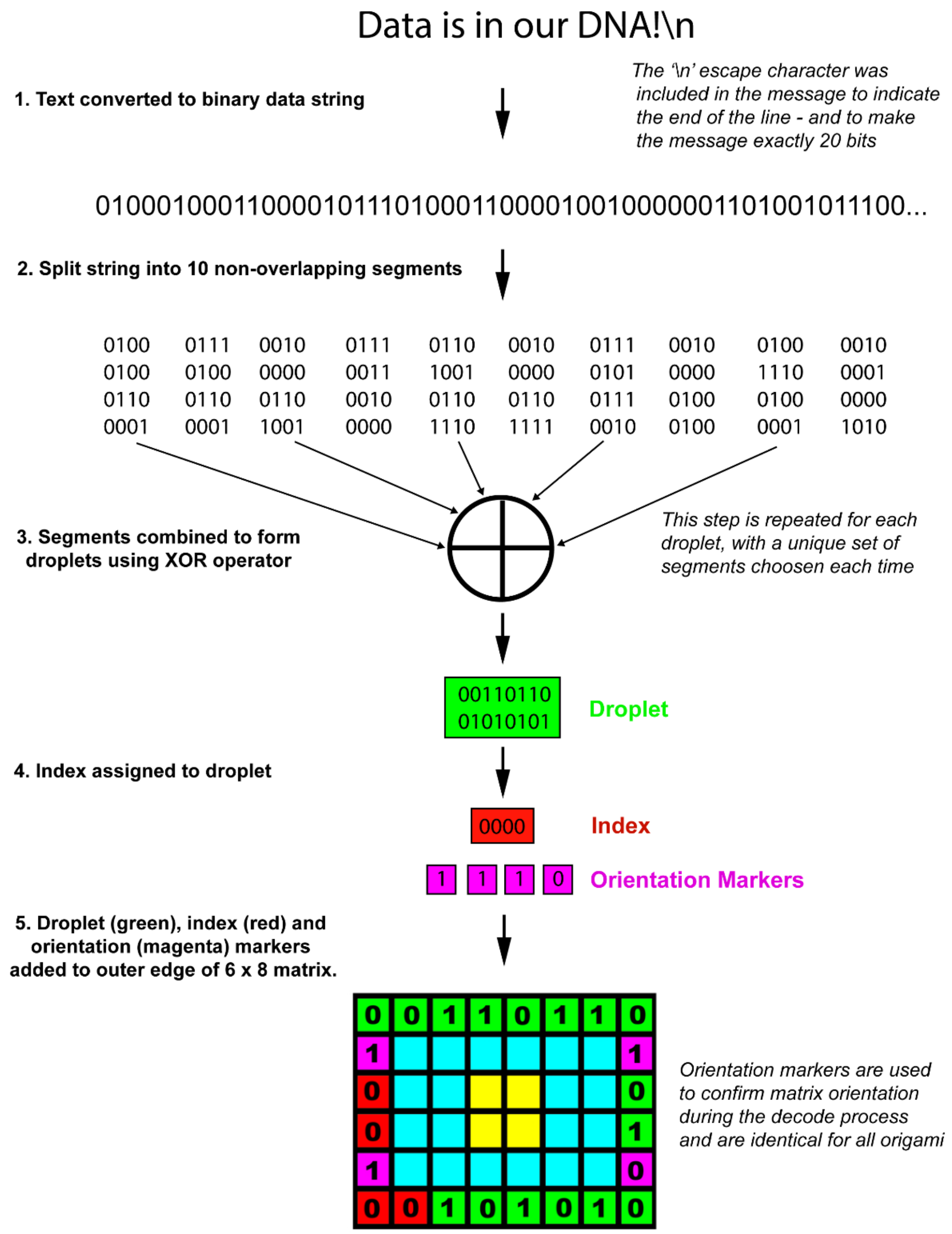

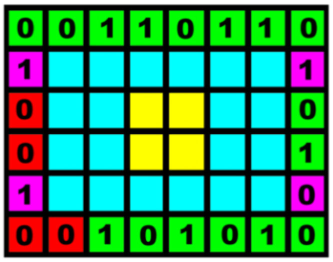

6. Checksum bits calculated from symetrically positioned matrix edge values

1	2	3	4	5	6	7	8
9	10	11	12	13	14	15	16
17	18	19	20	21	22	23	24
25	26	27	28	29	30	31	32
33	34	35	36	37	38	39	40
41	42	43	44	45	46	47	48

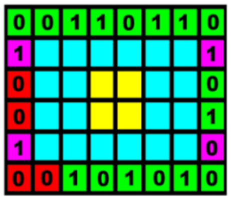

Bit values extracted by matrix position to generate checksum values

Matrix Position	1	2	3	4	5	6	7	8	9	16	17	24	25	32	33	40	41	42	43	44	45	46	47	48		XOR result
Binary Value	0	0	1	1	0	1	1	0	1	1	0	0	0	1	1	0	0	0	1	0	1	0	1	0		
	✓		✓							✓	✓									✓			✓		20	1
						✓		✓	✓			✓						✓			✓				21	1
				✓			✓						✓			✓	✓		✓						28	1
		✓			✓									✓	✓							✓		✓	29	0

Matrix position of result

7. Checksum bits added to center of matrix (yellow)

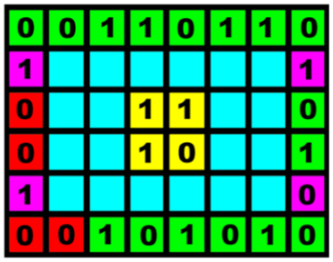

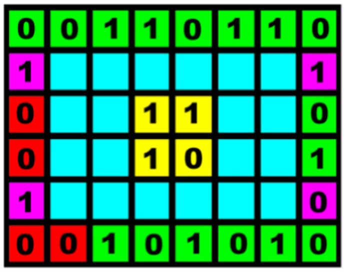

8. Parity bits calculated from symetrically positioned edge values and checksum

Matrix Position	1	8	41	48	2	7	42	47	43	46	3	6	4	5	44	45	9	16	33	40	17	24	25	32	20	21	28	29		XOR result
Binary Value	0	0	0	0	0	1	0	1	1	0	1	1	1	0	0	1	1	1	1	0	0	0	0	1	1	1	1	0		
	✓				✓							✓				✓									✓				10	1
		✓				✓					✓				✓											✓			15	1
			✓				✓			✓				✓													✓		34	1
				✓				✓	✓				✓															✓	39	1
					✓								✓				✓					✓				✓			11	1
						✓								✓				✓			✓				✓				14	1
							✓								✓				✓					✓				✓	35	0
								✓								✓				✓			✓				✓		38	1
	✓								✓		✓			✓										✓					12	1
		✓								✓		✓	✓										✓						13	0
			✓						✓		✓					✓						✓							36	1
				✓						✓		✓			✓						✓								37	1
	✓												✓					✓	✓		✓						✓		26	0
		✓												✓			✓			✓		✓						✓	31	1
			✓												✓		✓			✓			✓		✓				18	0
				✓												✓		✓	✓					✓		✓			23	1
	✓					✓			✓										✓				✓						27	1
		✓			✓					✓										✓				✓					30	1
			✓					✓			✓						✓				✓								19	1
				✓			✓					✓						✓				✓							22	0

Matrix position of result

This is similar to step 6, however the same values are combined in multiple differnet XOR operations

9. Parity bits added to remaining matrix positions (blue)

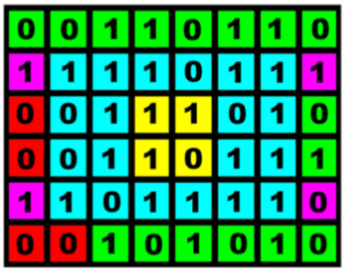

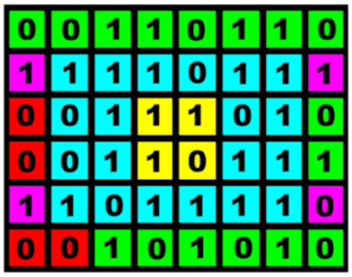

9. DNA-origami design created with the droplet, index, orientations markers, checksum bits, and parity bits encoded into the matrix

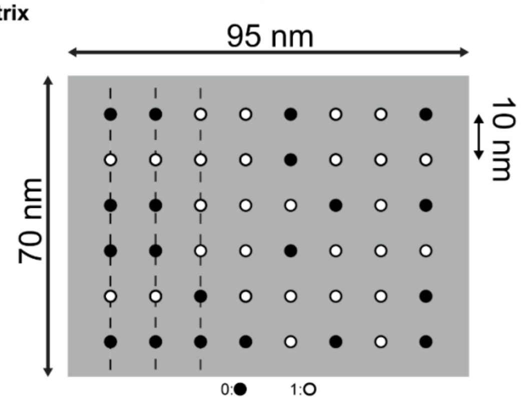

10. DNA-origami assembled from staple strands and scaffold in PCR thermocycler and purified by gel filtration

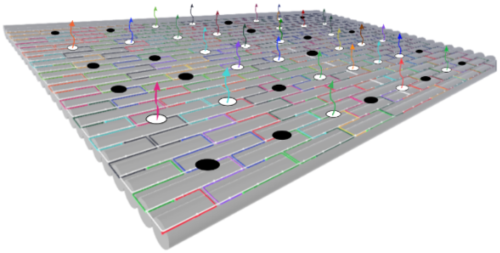

11. DNA-origami combined and stored at 4C

Fifteen differnt origami designs (one for each droplet) were used to encode the message 'Data is in our DNA!\n' . Each origami was diluted to 0.33 nM for storge.

4°C storage

4°C storage

12. DNA-origami tested using DNA-PAINT

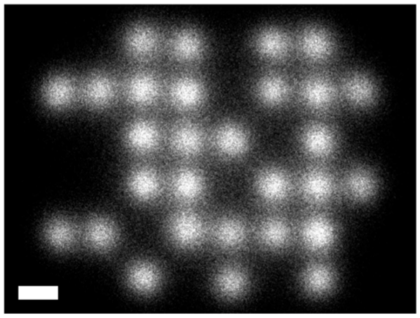

A small aliquot (1-2 ul) of the storage mix was diluted and deposited onto a microscope slide (using glow discharge) for DNA-PAINT imaging

An averaged image of origami-0 (the first of the fifteen designs) is shown here. Even though each of the individual single images from which the average is made is likely to be missing data from one or more data cells (due to data strands not correctly incorporating themselves into the origami structure, or insufficient imager strands binding to the data strand to get a 'read'), all the data cells are recorded correctly in the average. This suggests no systematic failure occured during the origami assembly. Similar averages were created for all fifteen origami designs, and all data cells in all origami were observed as expected.

Figure S5. The Decoding Algorithm

The main steps involved in decoding a message from dNAM are depicted. First, each individual origami captured in a DNA-PAINT recording is converted into a binary string (**Image Processing**). Next, errors in each binary string are detected and corrected if possible (**Error Correction**) using the algorithm described in Flowchart 1 (**SI Fig. S6**) and **index and droplet data extracted**. Finally, segment information is retrieved from the droplets (**Segment Information Extracted**) pooled with data from other origami and passed to the fountain code decoding algorithm shown in Flowchart 2 (**SI Fig. S6**), which reassembles the original file (**Fountain Code Decoding**).

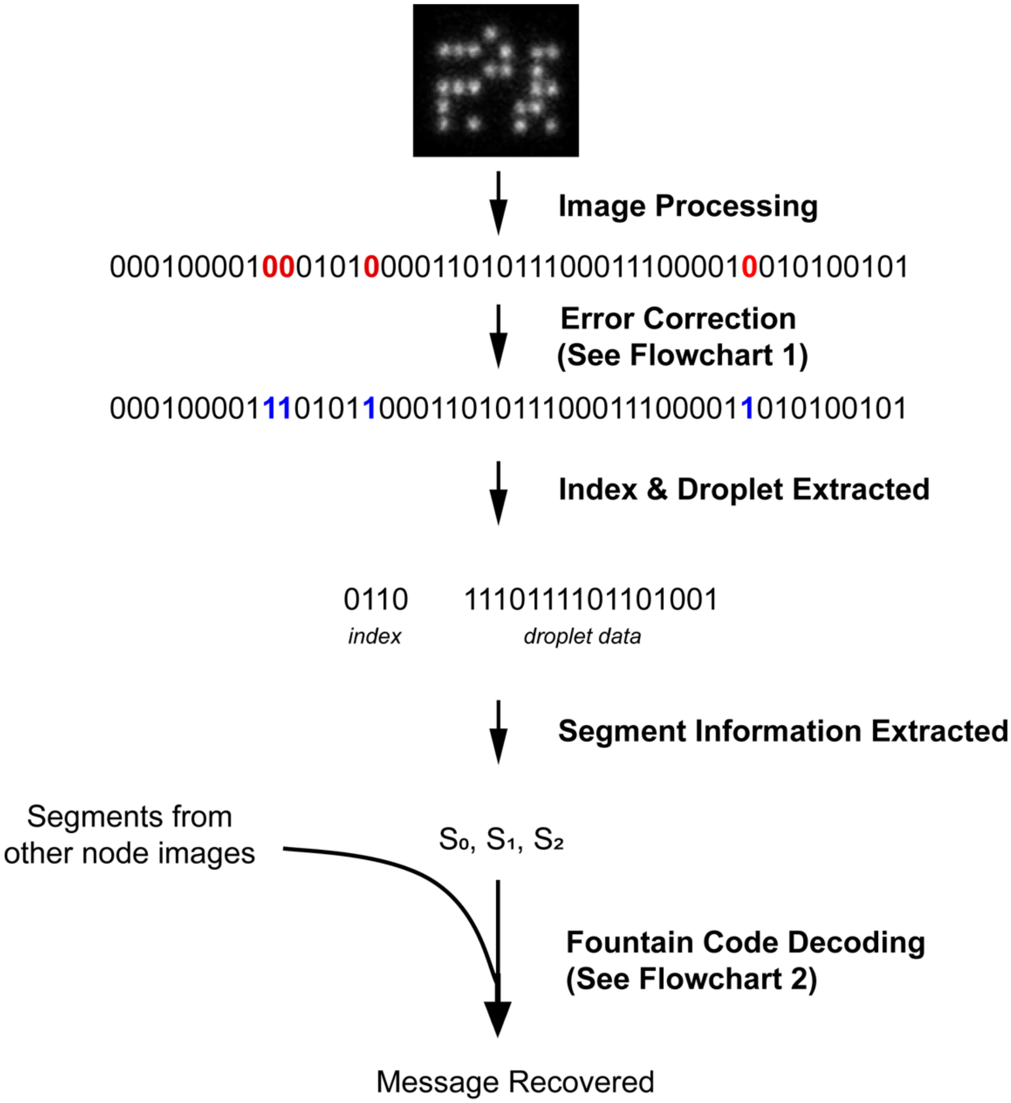

Figure S6. Flowchart 1 - Error Correction
A flowchart depicting the operations performed by the error correction algorithm for an individual origami is shown. A priority queue is initialized with an individual origami *m* (the *working_matrix*). Based on the parity and checksum bits mismatch, the algorithm deduces a set of probable errors and a matrix weight for the working matrix. The matrix weight is proportional to the number of errors, and the main goal of the algorithm is to reduce the matrix weight in a greedy fashion. To that end, each of the probable errors in the *working_matrix* is sequentially flipped, and a matrix weight calculated for every resulting matrix. The two resulting matrices with the lowest weights are enqueued. The algorithm then replaces the *working_matrix* with the recalculated matrix possessing the lowest matrix weight from the queue. If the current working matrix already has 9 bits flipped it is discarded and the next matrix in the queue used. The algorithm repeats these steps until the matrix weight equals zero, at this point the data in the origami is considered to have been error-corrected and is passed to the next stage of the decoding (**Accept**). If the priority queue is emptied before the matrix weight reaches zero, the origami data is considered unrecoverable and is removed from the analysis (**Reject**).

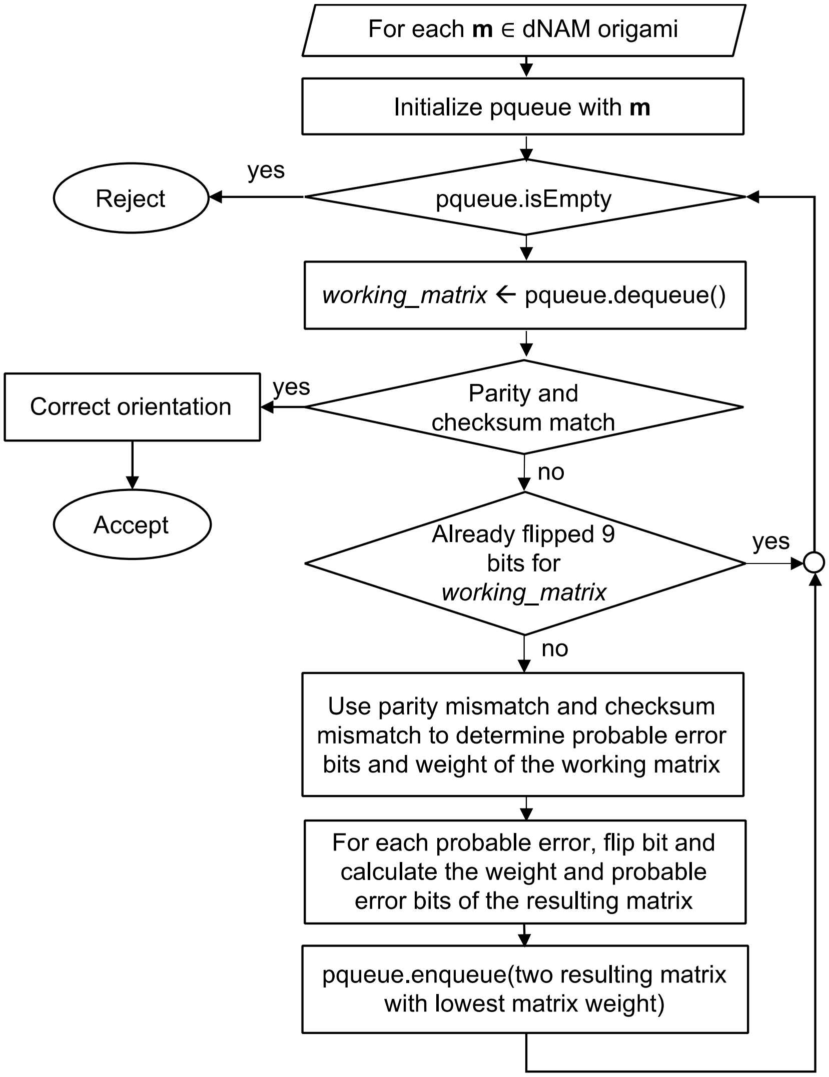

Figure S7. Flowchart 2 - Fountain Code Decoding

A flowchart demonstrating the operations performed by the fountain decoding algorithm to recover file segment data from droplet data is shown. After retrieving the binary data from the dNAM origami images, the dNAM decoding algorithm corrects errors in the binary data and extracts both the index and droplet information. The droplets are collected (**Droplet Table**), with each droplet containing one or more file segments. The data in single degree droplets, such as D_9 and D_8, encode single segments and are used directly to reconstruct the file (**Recovered File**).

To extract additional individual segment data from multi-segment droplets, the decoding algorithm performs a series of **XOR operations.** The index information allows the algorithm to determine both the degree of the droplet and which segments of the file that the droplet encodes. Taking the case of D_2, a series of XOR operations must be performed in order to retrieve additional segment data from it. The decoding algorithm may XOR a multi-degree droplet with another droplet if the other droplet's segment(s) are a proper subset of the multi-degree droplet. For example, the segments contained in D_6 are a proper subset of those in D_2. After XORing D_2 and D_6 a new droplet is generated containing segments S_5 and S_6, which ultimately leads to the algorithm extracting the data for S_6.

This process is repeated in a greedy fashion until the algorithm retrieves all of the file's segment data (**Recovered File**), or it runs out of options for XORing droplets (in which case the entire file cannot be successfully recovered). For simplicity, only six of the 15 possible droplets are shown, with the resulting recovered segments depicted in colored boxes (**Recovered Segments).**

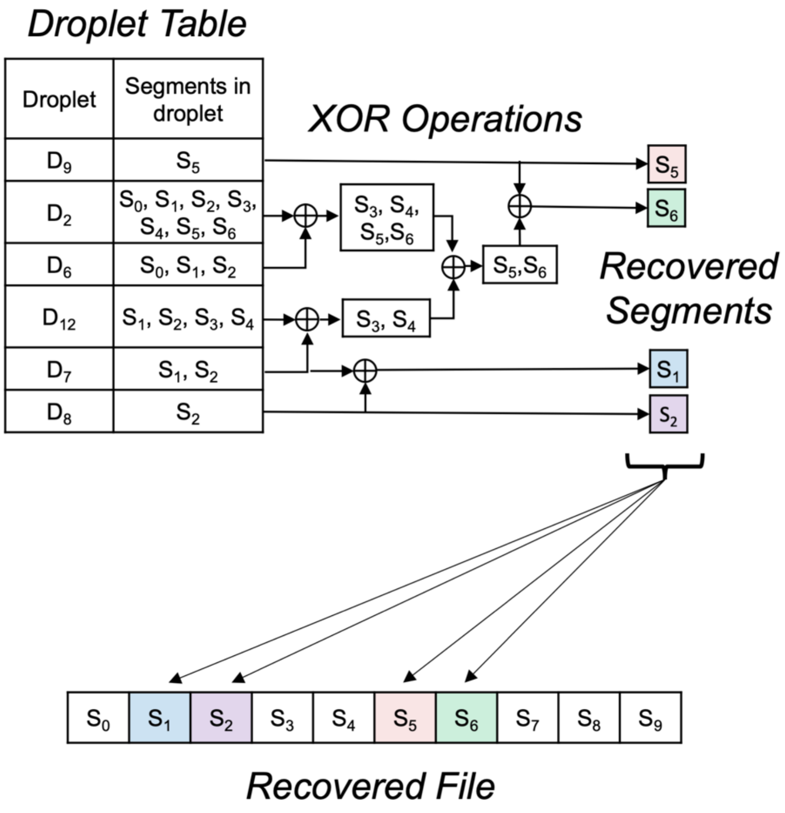

Figure S8. dNAM Summary

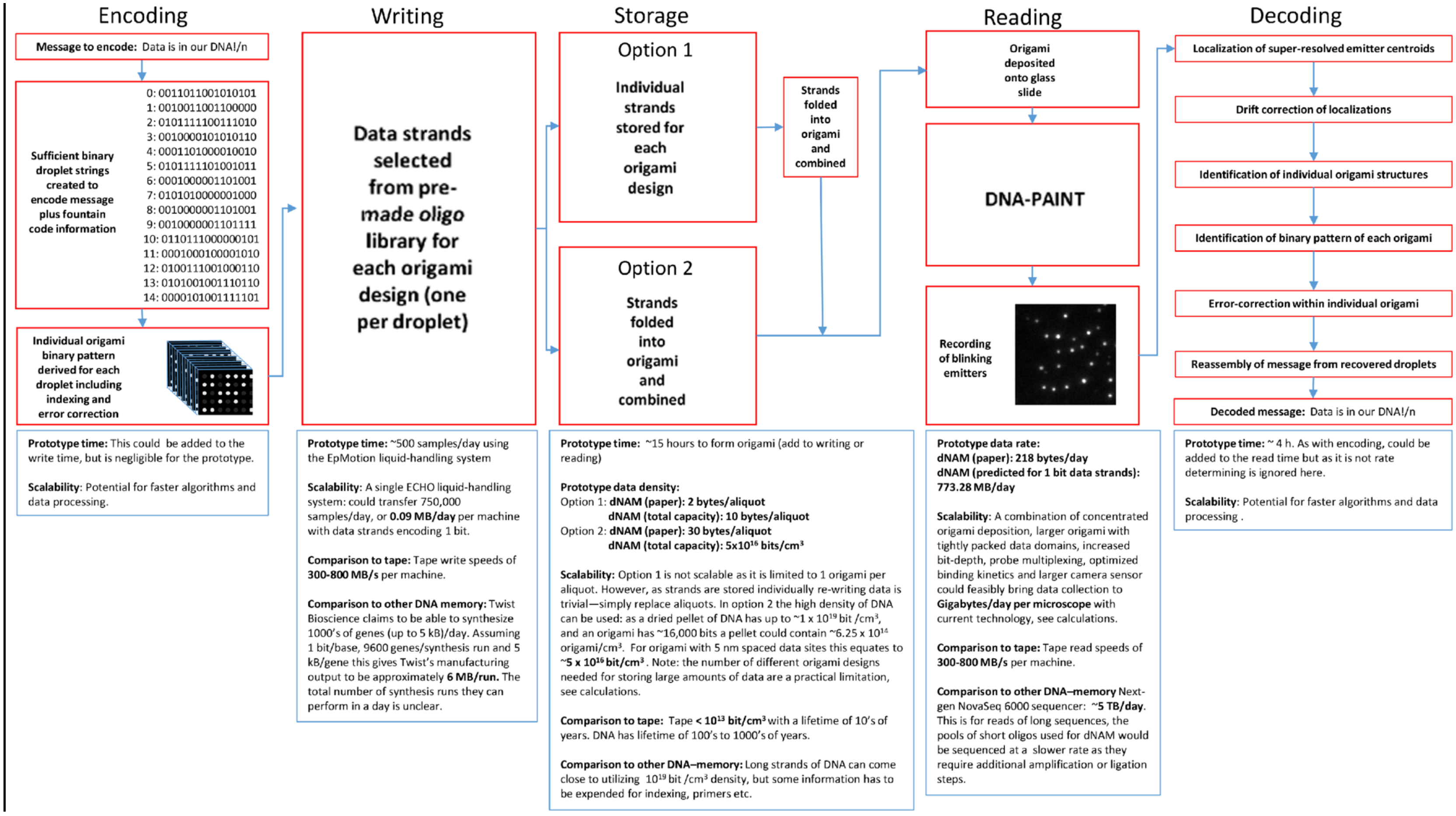

Supplementary Notes

Writing Speed

1. Echo liquid-handling system capacity
0.09 MB/day per machine (for writing dNAM prototype memory with 1 bit / domain)

Calculation

- From Labcyte website (https://www.labcyte.com/echo-liquid-handling) and documentation (Labcyte Inc. *Echo ® 520 Liquid Handler Technical Specifications Version 2.0*) an Echo can process ~750,000 samples/day.
- In the dNAM prototype there is one 1 data strand oligo per aliquot that needs to be transferred
- 1 data strand = 1 bit of stored information
- ∴750,000 data strand transfers = 750,000 bits/10^6 = 0.75 Mb
- 0.75 Mb / 8 = 0.0938 MB
  - ➔ **0.09 MB/day** per machine

2. TWIST Bioscience DNA synthesis
6 MB/day per synthesis run

We were not able to find exact values for long strands of DNA However, on their website Twist Bioscience claims to be able to synthesize 1000's of genes a day, up to 5 kB maximum length (also see Leproust 2016[5]). It isn't clear how many synthesis runs TWIST can actually perform per day (including QC etc.). However, as they only claim to be able to synthesize 1000's of genes/day, while each silicon chip can potentially handle 9,600 and is highly parallizable, it suggests their capacity could be considerably higher. As the global demand for synthetic DNA in 2015 was ~6 Gbases[6] (or ~750 MB) the estimated value may be in line with market demand.

Calculation

- For simplicity, assuming 1 bit/base (range of 0.19—1.71 bit/base for DNA-memory systems including primers, see Organick 2018) and 9600 genes (1 silicon chip's worth) per synthesis run:
- 9600 genes x 5000 bits = 48×10^6 bits = 48 Mb
- 48 Mb / 8 = 6 MB/synthesis run
  - ➔ **6 MB/day** per synthesis run

3. Magnetic Tape
300-800 MB/s per machine

(LTO-tape-drives, https://www.ibm.com/support/knowledgecenter/STQRQ9/com.ibm.storage.ts4500.doc/performance_specs_lto_drives.html)

Storage—Data Density

1. dNAM, unfolded data strands
Prototype: 2 bytes/aliquot

Total capacity (5 nm spaced data domains, no error-correction or indexing): 10 bytes/aliquot

For unfolded strands, in the dNAM prototype each origami design must be stored separately.

Calculations
dNAM prototype

- 1 data droplet (16 bits) per origami
  ➔ **2 bytes/aliquot**

Total capacity (5 nm spaced data domains, no error-correction or indexing)

The total capacity of a dNAM origami (without indexing, error-correction etc.) can be derived from the total number of bits available.

- For 5 x 5 nm spaced data domains on a rectangular platform there are 80 bits/origami
  ➔ **10 bytes/aliquot**.

Note: If indexing information is added to each strand, such that strands specific to an origami design could either be purified from a mixture, or self-assemble into the correct origami design, then data strands do not need to be stored separately. Storing them mixed would allow a dramatic increase in data density—perhaps getting close to the 10^{19} bit/cm^3 capacity of duplexed DNA[7].

2. dNAM, data strands folded into origami
Prototype: 30 bytes/aliquot

Total capacity (5 nm spaced data domains, no error-correction or indexing): $5x10^{16}$ bits/cm^3

After folding with scaffold DNA, origami can be stored mixed together increasing the amount of data per aliquot.

Calculations
dNAM prototype

- 15 data droplets (16 bits each) = 240 bits/aliquot
  ➔ **30 bytes/aliquot**

Total capacity (5 nm spaced data domains, no error-correction or indexing)

- A dried pellet of duplexed DNA has up to ~$1x10^{19}$ bit/cm^3 (from Zhirnov *et al*., 2016[7])
- A dNAM origami has ~16,000 bits total (including scaffold DNA and extended data strands)
- Assuming origami pack similarly to duplexed DNA, a pellet could contain ~$6.25x10^{14}$ origami/cm^3
- Origami with single bit data strands and 5 nm spacing have 80 bits/origami (without indexing, error-correction, etc.)
- 80 bits/origami x $6.25x10^{14}$ origami/cm^3 = $5x10^{16}$ bits/cm^3

➔ **$5x10^{16}$ bits/cm^3**

Note: While this storage capacity is significantly higher than magnetic tape, in practice a huge number of origami designs would need to be created to take full advantage of the available storage density. For example, with 80 bits/origami available, to store 1TB of data (no error-correction) 46% of the origami will need to be dedicated to indexing and ~$1x10^{11}$ different dNAM origami synthesized. Selecting the data strands alone would require ~$80x10^{11}$ selections from a library of premade oligonucleotides. With an Echo system this would take $2x10^{6}$ years. Another theoretical option could be to have all possible combinations of origami synthesized by a company like TWIST However, even then—at 6 MB/synthesis run it would take 166,666 runs to synthesize 1TB of DNA.

1. **DNA Storage capacity**

~$1x10^{19}$ bit/cm^3 (Zhirvnov *et al*., 2016[7])

2. **Magnetic Tape Capacity**

224 Gbit/in² (https://blocksandfiles.com/2020/06/29/fujifilm-400tb-magnetic-tape-cartridge-future/)

Calculation

- 224 Gbit/in² / 6.4516 = 34.7 Gb/cm^2
    - ➔ **$3.47x10^{10}$ bit/cm^2**

Reading Speeds

1. **dNAM/DNA-PAINT**
Prototype: 218 bytes/day per microscope

Prototype with 5 nm spaced data domains, dense deposition, 100 x speed-up and largest camera sensor available (total data, not considering error-correction, indexing and post-processing overhead): 773.28 MB/day per microscope

The latest report on DNA-PAINT by Strauss and Jungmann[8] describes a 100X speed-up in data collection for origami very similar to those we image in dNAM. 5 nm resolution was demonstrated with 100 ms camera integration times.

High deposition densities are feasible, especially as improved algorithms for processing dense SRM data have been, and continue to be developed (e.g. deepSTORM[9], DECODE[10]). deepSTORM is capable of processing data with a density of ~6 emitters/μm^2: with a 512x512 sensor of 2,982 μm^2 this is equivalent to ~17,900 emitters/frame.

A combination of concentrated origami deposition, larger origami with tightly packed data domains, increased bit-depth, probe multiplexing, optimized binding kinetics and larger camera sensor could feasibly bring data collection to **Gigabytes/day** per microscope with current technology.

Calculation
dNAM prototype

- 3.3 h for a 40,000 frame recording to recover 15 origami data (30 bytes)
- 30 bytes / 3.3 h
  - ➔ **218 bytes/day**

dNAM prototype with 5nm spaced data domains, 100 x speed-up, densely deposited origami and 1024 x 1024 camera sensor

- ~6 emitters/um^2 for 5964 μm^2 frame = 71600 emitters/frame
- For 5 nm spaced grid: ~80 binding events per site needed to achieve 5nm resolution (Schnitzbauer 2017)
- 71600 emitters/frame / 80 binding sites/site = 895 sites/frame
- 5 nm camera integration time = 0.1 s
- 1 site = 1 bit
- 8950 bits/s x 86400 s/day
  - ➔ **773.28 MB/day**

2. **NovaSeq 6000 sequencer**
~5 TB/day (https://www.illumina.com/science/technology/next-generation-sequencing.html)

This is for reads of long sequences, the pools of short oligonucleotides used for dNAM would be sequenced at a considerably slower rate as they require additional amplification or ligation steps

3. **Magnetic Tape**
300-800 MB/s

(LTO-tape-drives, https://www.ibm.com/support/knowledgecenter/STQRQ9/com.ibm.storage.ts4500.doc/performance_specs_lto_drives.html)

Supplementary References

1. Miller, E. J. *et al.* Sub-nanometer resolution imaging with amplitude-modulation atomic force microscopy in liquid. *J. Vis. Exp.* **2016**, 1–10 (2016).
2. Horcas, I. *et al.* WSXM: A software for scanning probe microscopy and a tool for nanotechnology. *Rev. Sci. Instrum.* **78**, (2007).
3. Strauss, M. T., Schueder, F., Haas, D., Nickels, P. C. & Jungmann, R. Quantifying absolute addressability in DNA origami with molecular resolution. *Nat. Commun.* **9**, 1–7 (2018).
4. Green, C. Nanoscale Optical and Correlative Microscopies for Quantitative Characterization of DNA Nanostructures. *Journal of Chemical Information and Modeling* vol. 53 (Boise State University, 2019).
5. Leproust, E. Rewriting DNA Synthesis. *Chem. Eng. Prog.* **112**, 30–35 (2016).
6. Organick, L. *et al.* Random access in large-scale DNA data storage. *Nat. Biotechnol.* **36**, 242–248 (2018).
7. Zhirnov, V., Zadegan, R. M., Sandhu, G. S., Church, G. M. & Hughes, W. L. Nucleic acid memory. *Nat. Mater.* **15**, 366–370 (2016).
8. Strauss, S. & Jungmann, R. Up to 100-fold speed-up and multiplexing in optimized DNA-PAINT. *Nat. Methods* **17**, 789–791 (2020).
9. Nehme, E., Weiss, L. E., Michaeli, T. & Shechtman, Y. Deep-STORM: super-resolution single-molecule microscopy by deep learning. *Optica* **5**, 458 (2018).
10. Matti, U., Obara, C. J., Wesley, R., Speiser, A. & Lucas-raphael, M. Deep learning enables fast and dense single-molecule localization with high accuracy. 1–28 (2020).
