# Supplementary material for: An alternative approach to nucleic acid memory
Source: Nat Commun. 2021 Apr 22;12:2371. doi: 10.1038/s41467-021-22277-y (PMC8062470; doi:10.1038/s41467-021-22277-y)
Supplement: Supplementary file 3 — Reporting Summary [file 41467_2021_22277_MOESM3_ESM.pdf]

nature research

Corresponding author(s): William L Hughes

Last updated by author(s): Mar 1, 2021

Reporting Summary

Nature Research wishes to improve the reproducibility of the work that we publish. This form provides structure for consistency and transparency in reporting. For further information on Nature Research policies, see our Editorial Policies and the Editorial Policy Checklist.

Statistics

For all statistical analyses, confirm that the following items are present in the figure legend, table legend, main text, or Methods section.

n/a	Confirmed	
☐	☒	The exact sample size (*n*) for each experimental group/condition, given as a discrete number and unit of measurement
☐	☒	A statement on whether measurements were taken from distinct samples or whether the same sample was measured repeatedly
☒	☐	The statistical test(s) used AND whether they are one- or two-sided<br>*Only common tests should be described solely by name; describe more complex techniques in the Methods section.*
☒	☐	A description of all covariates tested
☐	☒	A description of any assumptions or corrections, such as tests of normality and adjustment for multiple comparisons
☐	☒	A full description of the statistical parameters including central tendency (e.g. means) or other basic estimates (e.g. regression coefficient) AND variation (e.g. standard deviation) or associated estimates of uncertainty (e.g. confidence intervals)
☒	☐	For null hypothesis testing, the test statistic (e.g. *F*, *t*, *r*) with confidence intervals, effect sizes, degrees of freedom and *P* value noted<br>*Give P values as exact values whenever suitable.*
☒	☐	For Bayesian analysis, information on the choice of priors and Markov chain Monte Carlo settings
☒	☐	For hierarchical and complex designs, identification of the appropriate level for tests and full reporting of outcomes
☒	☐	Estimates of effect sizes (e.g. Cohen's *d*, Pearson's *r*), indicating how they were calculated

Our web collection on statistics for biologists contains articles on many of the points above.

Software and code

Policy information about availability of computer code

Data collection	Imaging data was collected using Nikon NIS-Elements version 5.20.00 and Princeton Instruments LightField version 5
Data analysis	Data analysis and simulations were performed using: ImageJ version 1.53c and the ThunderSTORM plugin version 1.3; Picasso-Render version 0.2.8; Python, version 3.7.3; OriginPro version 2019b; WSxM software package version 5.0 Develop 9.2; and custom code available on the GitHub repository (https://github.com/gmortuza/dnam)

For manuscripts utilizing custom algorithms or software that are central to the research but not yet described in published literature, software must be made available to editors and reviewers. We strongly encourage code deposition in a community repository (e.g. GitHub). See the Nature Research guidelines for submitting code & software for further information.

Data

Policy information about availability of data

All manuscripts must include a data availability statement. This statement should provide the following information, where applicable:

- Accession codes, unique identifiers, or web links for publicly available datasets
- A list of figures that have associated raw data
- A description of any restrictions on data availability

The original DNA-PAINT recordings and drift-corrected centroid localization data that support the findings of this study have been deposited in the Zenodo repository with the identifier “doi: 10.5281/zenodo.4546134”.

Field-specific reporting

Please select the one below that is the best fit for your research. If you are not sure, read the appropriate sections before making your selection.

☒ Life sciences ☐ Behavioural & social sciences ☐ Ecological, evolutionary & environmental sciences

For a reference copy of the document with all sections, see nature.com/documents/nr-reporting-summary-flat.pdf

Life sciences study design

All studies must disclose on these points even when the disclosure is negative.

Sample size	No sample-size calculation performed. As a proof-of-principle, we feel only limited numbers of repeats are required to show that data can be encoded and retrieved and further experiments testing origami stability, probe kinetics or algorithm performance would be more appropriate than increasing the n number for the experiments described here (and are underway).
Data exclusions	No data were excluded.
Replication	Each of the 15 DNA-origami used in the experiments was synthesized separately (two times each), combined and imaged three times to determine if the encoded message could be recovered. Several of the origami were also repeatedly synthesized for internal quality control (data not included in the manuscript). All attempts at replication were successful.
Randomization	Randomization of sample selection was carried out at two points in our analysis. 1) Origami were randomly selected from the imaged field-of-view during localization data processing; 2) A random subsection of decoded messages were sampled to determine the number of origami needed to decode a message.
Blinding	Blinding not performed. Origami data collection was automated and no blinding required.

Reporting for specific materials, systems and methods

We require information from authors about some types of materials, experimental systems and methods used in many studies. Here, indicate whether each material, system or method listed is relevant to your study. If you are not sure if a list item applies to your research, read the appropriate section before selecting a response.

Materials & experimental systems

n/a	Involved in the study
☒	☐ Antibodies
☒	☐ Eukaryotic cell lines
☒	☐ Palaeontology and archaeology
☒	☐ Animals and other organisms
☒	☐ Human research participants
☒	☐ Clinical data
☒	☐ Dual use research of concern

Methods

n/a	Involved in the study
☒	☐ ChIP-seq
☒	☐ Flow cytometry
☒	☐ MRI-based neuroimaging
