# Supplementary material for: An alternative approach to nucleic acid memory
Source: Nat Commun. 2021 Apr 22;12:2371. doi: 10.1038/s41467-021-22277-y (PMC8062470; doi:10.1038/s41467-021-22277-y)
Supplement: Supplementary file 2 — Peer Review File [file 41467_2021_22277_MOESM2_ESM.pdf]

Editorial Note: This manuscript has been previously reviewed at another journal that is not operating a transparent peer review scheme. This document only contains reviewer comments and rebuttal letters for versions considered at *Nature Communications*. Mentions of prior referee reports have been redacted.
